# Genomic Identification and Comparative Expansion Analysis of the *Non-Specific Lipid Transfer Protein* Gene Family in *Gossypium*

**DOI:** 10.1038/srep38948

**Published:** 2016-12-15

**Authors:** Feng Li, Kai Fan, Fanglu Ma, Erkui Yue, Noreen Bibi, Ming Wang, Hao Shen, Md Mosfeq-Ul Hasan, Xuede Wang

**Affiliations:** 1 Institute of Crop Science, College of Agriculture and Biotechnology, Zhejiang University, Hangzhou 310058, People’s Republic of China; 2 College of Crop Science, Fujian Agriculture and Forestry University, Fuzhou, 350002, Fujian, China; 3 Nuclear Institute for Agriculture and Biology, Faisalabad, Pakistan

## Abstract

Plant non-specific lipid transfer proteins (nsLTPs) are involved in many biological processes. In this study, 51, 47 and 91 *nsLTPs* were identified in *Gossypium arboreum, G. raimondii* and their descendant allotetraploid *G. hirsutum*, respectively. All the *nsLTPs* were phylogenetically divided into 8 distinct subfamilies. Besides, the recent duplication, which is considered cotton-specific whole genome duplication, may have led to *nsLTP* expansion in *Gossypium*. Both tandem and segmental duplication contributed to *nsLTP* expansion in *G. arboreum* and *G. hirsutum*, while tandem duplication was the dominant pattern in *G. raimondii*. Additionally, the interspecific orthologous gene pairs in *Gossypium* were identified. Some *GaLTPs* and *GrLTPs* lost their orthologs in the A_t_ and D_t_ subgenomes, respectively, of *G. hirsutum*. The distribution of these *GrLTPs* and *GaLTPs* within each subfamily was complementary, suggesting that the loss and retention of *nsLTPs* in *G. hirsutum* might not be random. Moreover, the *nsLTPs* in the A_t_ and D_t_ subgenomes might have evolved symmetrically. Furthermore, both intraspecific and interspecific orthologous genes showed considerable expression variation, suggesting that their functions were strongly differentiated. Our results lay an important foundation for expansion and evolutionary analysis of the *nsLTP* family in *Gossypium*, and advance *nsLTP* studies in other plants, especially polyploid plants.

Lipids play a vital role in plant growth and development. They can maintain cell function and mediate cell signaling associated with stress responses. Plant non-specific lipid transfer proteins (nsLTPs) (6.5–10.5 kDa in size) are able to transfer phospholipids and fatty acids between membranes *in vitro*^1^,^2^. They are abundantly present in various plants, representing up to 4% of the total soluble protein^2^. These peptides are structurally characterized by an eight cysteine motif (8 CM) backbone with the general form C-Xn-C-Xn-CC-CXC-Xn-C-Xn-C, and the cysteine residues are linked by four disulfide bonds to stabilize a tertiary structure of a hydrophobic cavity^2^,^3^. Moreover, almost all nsLTPs carry an N-terminal secretory signal peptide (21–27 amino acids in length) in their nascent polypeptides, indicating that they are secreted proteins^2^,^4^. In previous studies, plant nsLTPs have been identified to participate in a range of biological processes, including cuticular wax and cutin synthesis, biotic and abiotic stress resistance, plant signaling, seed maturation and sexual reproduction^2^,^4^. In some cases, nsLTPs are considered pathogenesis-related (PR) proteins and constitute the PR-14 family^5^. The defensive role of plant nsLTPs against fungal, bacterial and viral pathogens has been well verified by ample evidence^2^,^6^,^7^,^8^.

Plant nsLTPs have been well studied and identified in many plant species including both flowering and non-flowering plants^2^. They were initially classified into two types according to molecular mass: nsLTP1 (9 kDa) and nsLTP2 (7 kDa)^1^. The disulfide bond linkages of nsLTP1 at Cys_1_-Cys_6_ and Cys_5_-Cys_8_ differ from those of nsLTP2 at Cys_1_-Cys_5_ and Cys_6_-Cys_8_. Recently, a new classification according to sequence similarity and intervals of 8 CM was proposed by Boutrot *et al*.^3^. The system categorized 267 *nsLTPs* into nine types (Type I-IX) based on a genome-wide analysis of rice (*Oryza sativa*), wheat (*Triticum aestivum*) and *Arabidopsis thaliana*. Then, *nsLTPs* in other plant species were also grouped according to Boutrot’s method with slight modifications^4^,^9^,^10^,^11^,^12^, and novel types such as Type X^9^ and Type XI^4^ were identified.

Cotton provides the world’s most utilized natural fiber for the textile and garment industries. The genus *Gossypium* comprises approximately 45 diploid species and can be divided into eight monophyletic groups (each designated alphabetically as A through G, and K)^13^,^14^. The A- and D- genome diploids diverged from the same eudicot progenitor approximately 5–10 million years ago (MYA). Then, ancient hybridization between A and D diploids occurred, resulting in the generation of a clade of five allotetraploid species approximately 1–2 MYA^15^. *Gossypium hirsutum* is one of the descendant allotetraploid species and may be derived from a spinnable fiber capable A genome species (*G. arboreum*) and a non-spinnable fiber capable D genome species (*G. raimondii*)^15^,^16^. Cotton polyploidization has altered the function of the A and D ancestral diploid genomes and conferred emergent properties^17^. More than 90% of world cotton production is attributable to the cultivation of *G. hirsutum*^15^. However, a soil-borne vascular disease, *Verticillium* wilt, which is caused by *Verticillium dahliae*, is the most prevalent and lethal disease affecting cotton production. It is a critical plant quarantine disease and called the “cancer” of cotton crop^18^. Few *G. hirsutum* germplasms were found to be resistant to *V. dahliae*^19^. Comprehensive prevention and control measures are limited solely to improvements in genetic resistance^19^,^20^.

In previous studies, only a small portion of nsLTPs from cotton have been characterized^2^,^21^,^22^. With *G. raimondii, G. arboreum* and *G. hirsutum* genome sequencing completed^14^,^15^,^23^, an excellent opportunity is coming to initiate whole-genome annotation and to perform comparative genomic study in *Gossypium*. Until now, a genome-wide overview of the *nsLTP* family in *Gossypium* has yet to be reported. Thus, a systematic molecular evolution and expansion analysis of the *nsLTPs* in *Gossypium* is urgently required. In this study, putative *nsLTPs* were identified in *G. raimondii, G. arboreum* and *G. hirsutum*. We conducted a comprehensive study on the phylogenetics, genomic structure, chromosomal localization and expansion history to evaluate the molecular evolution, expansion history and expression profiles of the *nsLTP* family in *Gossypium*.

## Results

### Genomic identification of putative *nsLTPs* in *G. raimondii, G. arboreum* and *G. hirsutum*

The availability of *G. raimondii, G. arboreum* and *G. hirsutum* genome sequences makes it possible to identify all the *nsLTPs* in the three *Gossypium* species. The BLASTP program was utilized to search for candidate *nsLTPs* in cotton with the query sequences from Arabidopsis. Initially, 104, 104 and 182 protein sequences were identified in *G. raimondii, G. arboreum* and *G. hirsutum*, respectively. After screening and selection as described in the materials and methods, a total of 189 *nsLTPs* were confirmed and described (Table S2). Among them, *G. raimondii* and *G. arboreum* contain a similar number of *nsLTPs* (47 and 51, respectively), despite the fact that *G. raimondii* has a much smaller genome size (885 Mb/1 C) than *G. arboreum* (1,746 Mb/1 C). In *G. hirsutum* (2,173 Mb), 91 *nsLTPs* were identified, representing almost a two-fold increase over the number of *nsLTPs* in its diploid progenitors. We designated the genes identified in *G. raimondii, G. arboreum* and *G. hirsutum* as *GrLTPs, GaLTPs* and *GhLTPs*, respectively.

### Phylogenetic analysis of the *nsLTP* family

**Figure 1:**
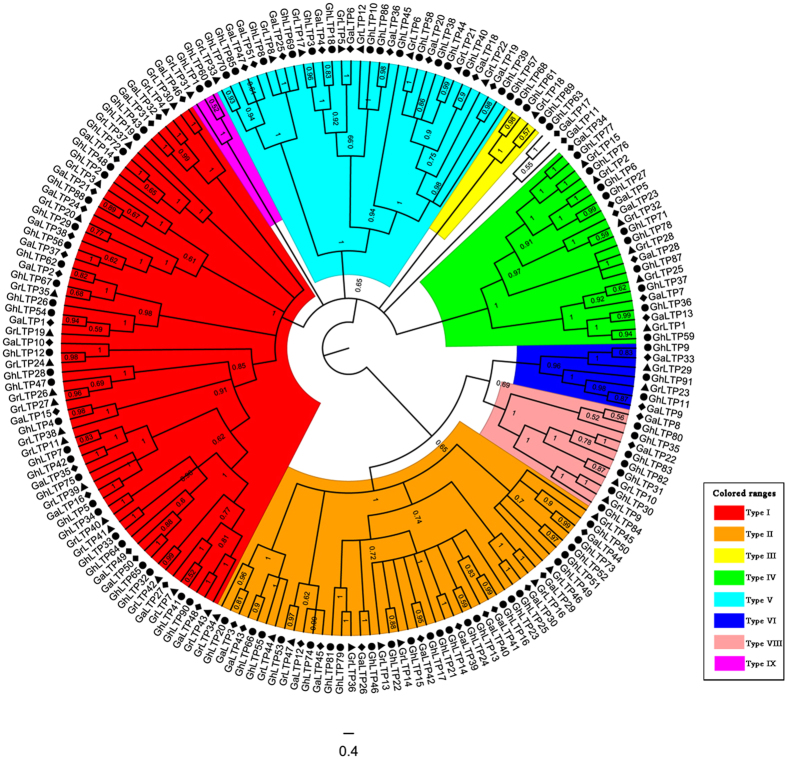
Phylogenetic relationships of the *nsLTP* family from *G. raimondii, G. arboreum* and *G. hirsutum*. The phylogenetic tree was generated using the Bayesian method based on multiple alignments of the nsLTP sequences. The numbers in the clades are posterior probability values. The *nsLTP* subfamilies are indicated by different colors.

To determine the evolutionary relationships of *nsLTPs*, phylogenetic analysis of the identified *nsLTPs* in *Gossypium* and Arabidopsis was completed with the MrBayes and PHYLIP tools (Fig. 1, Fig. S1). There were similar results with high support values from each method. According to Boutrot’s classification system, the *nsLTP* family in *Gossypium* was divided into 8 subfamilies (Type I, II, III, IV, V, VI, VIII and IX), and no Type VII *nsLTPs* were identified in cotton (Fig. 1, Fig. S1, Table S2). The member proportion was different in each subfamily (Fig. S2a). The Type I subfamily (33.33%) contained the most members, followed by Type II (23.28%), Type V (16.93%) and Type IV (11.64%). The least represented subfamily was Type IX (1.59%). A similar member distribution in each subfamily was found in each *Gossypium* species (Fig. S2b). Besides, the proportion of *nsLTPs* in Type I was 35.29% and 38.30% in *G. arboreum* and *G. raimondii*, respectively, whereas it decreased to 29.67% in *G. hirsutum*. The proportion of members in both Type II and Type VIII in *G. hirsutum* (25.27% and 7.69%, respectively) was higher than that in *G. arboreum* (23.53% and 3.92%, respectively) and *G. raimondii* (19.15% and 4.26%, respectively). Moreover, not all the subgroups were present in each *Gossypium* species, and no Type III and Type IX *nsLTPs* existed in *G. arboreum*.

Additionally, all the 189 nsLTP sequences were evaluated with OrthoMCL clustering. With the default stringency, 21 orthologous groups (OGs) in 8 subfamilies were shown (Table S3). Each subfamily contained one or more OGs, and each distinct OG was shared by only one specific subfamily. The OG distributions were similar to the phylogenetic classifications of the *nsLTP* family in the three *Gossypium* species. The protein structures were highly diverse in all the identified nsLTPs (Table S1). The amino acid lengths of the nsLTPs in the Type I, Type V and Type VIII subfamilies were relatively longer, while the proteins in Type II and Type III had relatively shorter amino acid lengths. A similar distribution in the molecular weight of the nsLTPs also existed.

### Conserved protein motifs and exon/intron structure of *nsLTPs*

**Figure 2:**
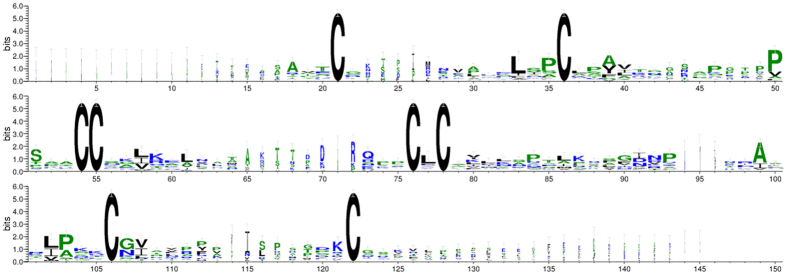
Conserved domain analysis of the nsLTPs using the WebLogo program. The height of the letter designating the amino acid residue at each position represents the degree of conservation. The numbers on the x-axis represent the sequence positions in the corresponding conserved domains. The y-axis represents the information content measured in bits.

The main characteristic of plant nsLTPs is the presence of 8 CM in highly conserved positions. Using the WebLogo program, the sequence logos of the identified nsLTPs in *Gossypium* were generated to further confirm the conservation of amino acid residues (Fig. 2). Moreover, a variable number of inter-cysteine amino acid residues was displayed through multiple alignments, and 8 nsLTP types were therefore identified based on the typical spacing of the 8 CM (Table 1). Between the conserved Cys_1_ and Cys_2_ residues, Type II and VIII nsLTPs contained 6–8 residues, while Type V and IX contained 14 and 13 residues, respectively. Between the conserved Cys_4_ and Cys_5_ residues, Type I nsLTPs contained 19-20 residues, while the other types contained relatively fewer residues (8–12). Between the conserved Cys_6_ and Cys_7_ residues, Type III nsLTPs contained 12 residues, while the other types contained more residues (19–27). Between the conserved Cys_7_ and Cys_8_ residues, Type I nsLTPs contained more residues (13–15) than Type III and Type IX nsLTPs (6).

**Figure 3:**
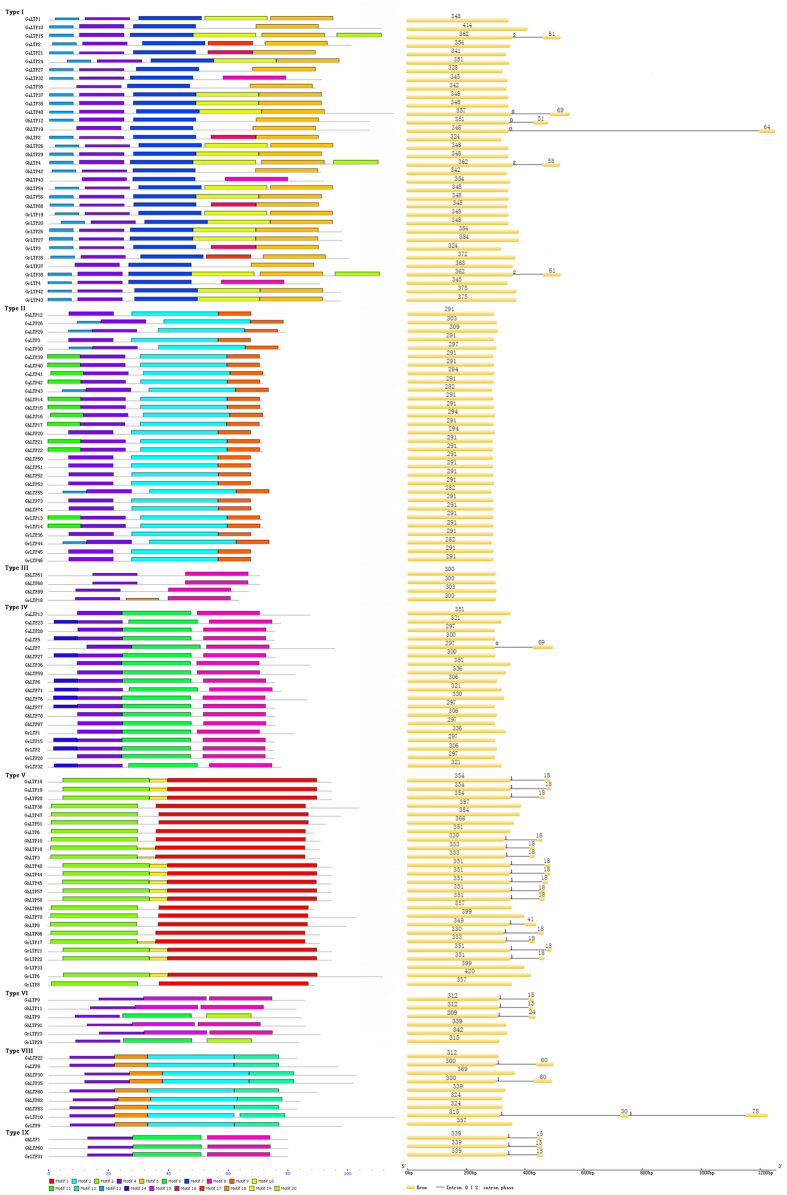
Motif compositions (left panel) and gene structures (right panel) of some nsLTP members in *Gossypium*. The conserved domains were identified using the MEME web server. Different motifs are represented by various colored boxes. The location of each motif can be estimated using the scale at the bottom. Gene structures of *nsLTPs* were predicted with the GSDS software. The exons are indicated by yellow boxes and the introns are indicated by black lines.

The MEME motif search tool was employed to identify 20 distinct conserved motifs in the nsLTPs (Fig. 3). Based on the distribution of the predicted motifs, the 189 nsLTPs were categorized into 8 distinct subfamilies, which was consistent with the classification from the phylogenetic analysis. All the nsLTPs had either Motif 4 or 3, which represented Cys_2_. Motif 13, 11 and 14 corresponded to Cys_1_, and Motif 7, 18, 19, 6 and 15 mapped to Cys_3_Cys_4_. In addition, Motif 8, 9, 5, 1 and 12 corresponded to Cys_7_ and Cys_8_. Motif 1, 2, 6, 10 and 15 mapped to Cys_5_XCys_6_, and different residues were found in the central position of the Cys_5_XCys_6_ motif. Seven hydrophilic residues (Arg, Gly, Glu, Asp, Gln, Ser and Lys) and five hydrophobic residues (Tyr, Phe, Leu, Val and Met) existed at the X position of the Cys_5_XCys_6_ motif in the 189 nsLTPs. Moreover, some subfamily-specific motifs were identified. For instance, Motif 5, 7 and 10 were only present in Type I nsLTPs, and Motif 15 only existed in Type VI nsLTPs.

The gene structure of the *nsLTP* family was also investigated (Fig. 3). Our results revealed low diversity in the distribution of intronic regions amid the exonic sequences. The intron patterns, formed by relative position and phase, were highly conserved within each phylogenetic group. The number of introns per gene varied from 0 to 2. No introns were identified in Type II and Type III *nsLTPs*, while some *nsLTPs* in other subfamilies were interrupted by 1–2 introns positioned 15 to 78 bp downstream of the codon, which almost encoded the Cys_8_ in the 8 CM.

### Chromosomal localization and duplication of *nsLTPs* in cotton

**Figure 4:**
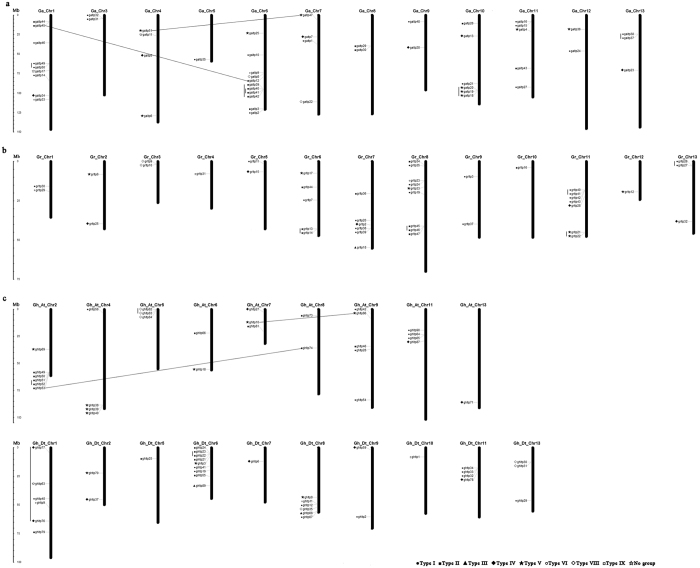
Chromosomal locations of *nsLTPs* from *G. arboreum* (a) *G. raimondii* (b) and *G. hirsutum* (c). The scale represents megabases (Mb). Chromosome numbers are indicated above each vertical bar. The markers before the gene names indicate the *nsLTP* subfamily. The duplicated gene pairs are joined by black lines.

Based on the genomic location of *nsLTPs* (Table S2), chromosomal distribution diagrams of the *nsLTP* family were generated for the three *Gossypium* species (Fig. 4). In *G. arboreum*, the 51 *nsLTPs* were distributed unevenly on 12 chromosomes, and the number of *nsLTPs* on each chromosome varied widely (Fig. 4a). Chromosome 6 had the largest number of *nsLTPs* with 11 members, followed by Chromosome 1 with 9 genes. In contrast, only 1 gene was located on Chromosome 5, and no *nsLTPs* were found in Chromosome 2. In *G. raimondii*, the 43 *nsLTPs* were located on 13 chromosomes, with Chromosome 8 containing the largest number of genes (9) (Fig. 4b). Two genes were present on Chromosome 1, 2, 3, 5 and 9, whereas only a single *nsLTP* was localized on Chromosome 4, 10 and 12 each. In addition, a total of 30 and 35 *nsLTPs* were mapped in the 9 A_t_ and 10 D_t_ subgenome chromosomes of *G. hirsutum*, respectively (Fig. 4c). D_t_- Chromosome 6 contained the maximum number of *nsLTPs* with 9 genes, followed by A_t_- Chromosome 2, D_t_- Chromosome 1 and D_t_- Chromosome 8 with 6 genes each, while only one gene was present on A_t_- Chromosome 13, D_t_- Chromosome 5, D_t_- Chromosome 7 and D_t_- Chromosome 10 each. Besides, no *nsLTPs* were located on A_t_- Chromosome 1, 3, 10 and 12, and D_t_- Chromosome 3, 4 and 12. Moreover, several *nsLTP* clusters were detected on chromosomes such as the top of D_t_-Chromosome 6 and the bottom of A_t_-Chromosome 2 in *G. hirsutum*.

Gene duplication events were investigated to elucidate the expansion pattern of the *nsLTP* family in *Gossypium*. In our study, 7, 5 and 6 duplicated gene pairs were identified in *G. arboreum, G. raimondii* and *G. hirsutum*, respectively (Table 2). In *G. arboreum* and *G. raimondii*, the duplication events were concentrated in similar subfamilies (including Type I, II and V), whereas in *G. hirsutum* the duplication events occurred in Type II, IV, V and VIII. This result revealed the strong expansion preference for some *nsLTP* subfamilies in *Gossypium*. Meanwhile, based on the sequence analysis and the chromosomal distribution, 2 and 2 paired genes in *G. arboreum* and *G. hirsutum*, respectively, were identified to be involved in segmental duplication events, while the other 14 pairs were linked to tandem duplication events.

In addition, the Ka/Ks ratio of each duplicated gene pair was calculated to assess the molecular evolutionary rates (Table 2). In this study, 83.3% of the duplicated *nsLTPs* from cotton have mainly undergone purifying selection pressure after the duplication events (Table 2). As purifying selection apparently constrains the divergence of the duplicated genes, the functions of the duplicated *nsLTPs* might not diverge much during subsequent evolution. Moreover, the divergence times between the duplicated gene pairs were analyzed. In *G. arboreum* and *G. raimondii*, most of the Ks values were less than 0.17, and their corresponding duplication age might be less than 32.69 million years ago (MYA). Only one duplication event (*GaLTP47*/*51*) occurred approximately 194.74 MYA. In *G. hirsutum*, the Ks values were 0.03–0.13, and the duplication ages were estimated to be 5.84–24.50 MYA.

### Orthologous gene analysis and synteny block detection

**Figure 5:**
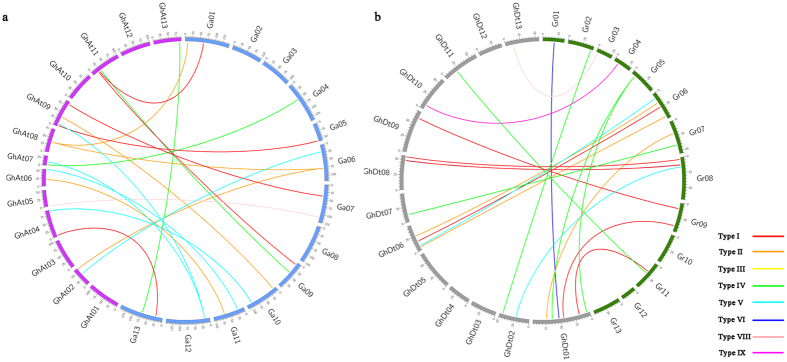
Distribution of *nsLTP* orthologous gene pairs and synteny block identification between the A_t_ subgenome in *G. hirsutum* and the diploid A genome in *G. arboreum* genome (a) and the D_t_ subgenome in *G. hirsutum* and the diploid D genome in *G. raimondii* (b). The differently colored lines represent the subfamilies within the *nsLTP* family.

The evolution of *nsLTPs* was analyzed among the three *Gossypium* species. We identified 23 and 24 orthologous gene pairs within the A_t_ and D_t_ subgenomes, respectively, in *G. hirsutum* and their corresponding ancestral A and D diploid genomes (Fig. 5, Table S4). Of the orthologous gene pairs, most were distributed in Type I, II, IV and V (Fig. S3, Table S4). Besides, 24 *GrLTPs* have no orthologs in the D_t_ subgenome of *G. hirsutum*, and 31 *GaLTPs* have no orthologs in the A_t_ subgenome. Meanwhile, the Ks values of the orthologous gene sets (A_t_ vs A and D_t_ vs D) were also compared. The divergence times between the allotetraploid and its progenitor genomes were estimated to be 3.46–3.54 MYA (Ks peaks at 0.0180 and 0.0184, respectively) (Fig. S4). The similar divergence time suggested that the *nsLTPs* in A_t_ and D_t_ subgenomes might have evolved symmetrically.

The conservation and rearrangement of certain chromosome segments may play an important role in the adaptive evolution of the three *Gossypium* species. In our study, we discovered 3 conserved syntenic blocks between the A_t_ subgenome in *G. hirsutum* and the A diploid genome in *G. arboreum*, and 1 conserved syntenic block between the D_t_ subgenome in *G. hirsutum* and the D diploid genome in *G. raimondii*. This result indicated that the collinear relationships of the *nsLTPs* between the A_t_ subgenome and the *G. arboreum* genome were higher than those between the D_t_ subgenome and the *G. raimondii* genome. These conserved segments contain different numbers of *nsLTPs*, ranging from 2 to 3. According to the analysis, the genes situated on *G. hirsutum* A_t_ Chromosome 11, 4 and 5 were predicted to have corresponding orthologs on *G. arboreum* Chromosome 1, 10 and 7, respectively, whereas the genes on *G. hirsutum* D_t_ Chromosome 11 were found to originate from the conserved block on *G. raimondii* Chromosome 11 (Fig. 5). In particular, six paired *nsLTPs* (*GhLTP64*/*GaLTP49, GhLTP65*/*GaLTP50, GhLTP38*/*GaLTP20, GhLTP39*/*GaLTP19, GhLTP82*/*GaLTP22* and *GhLTP83*/*GaLTP22*) were located in genomic regions with synteny between the *G. hirsutum* and *G. arboreum* genomes, while three paired *nsLTPs* (*GhLTP32*/*GrLTP42, GhLTP33*/*GrLTP41* and *GhLTP34*/*GrLTP40*) were located in genomic regions with synteny between the *G. hirsutum* and *G. raimondii* genomes.

### Analysis of orthologous gene expression patterns in cotton

**Figure 6:**
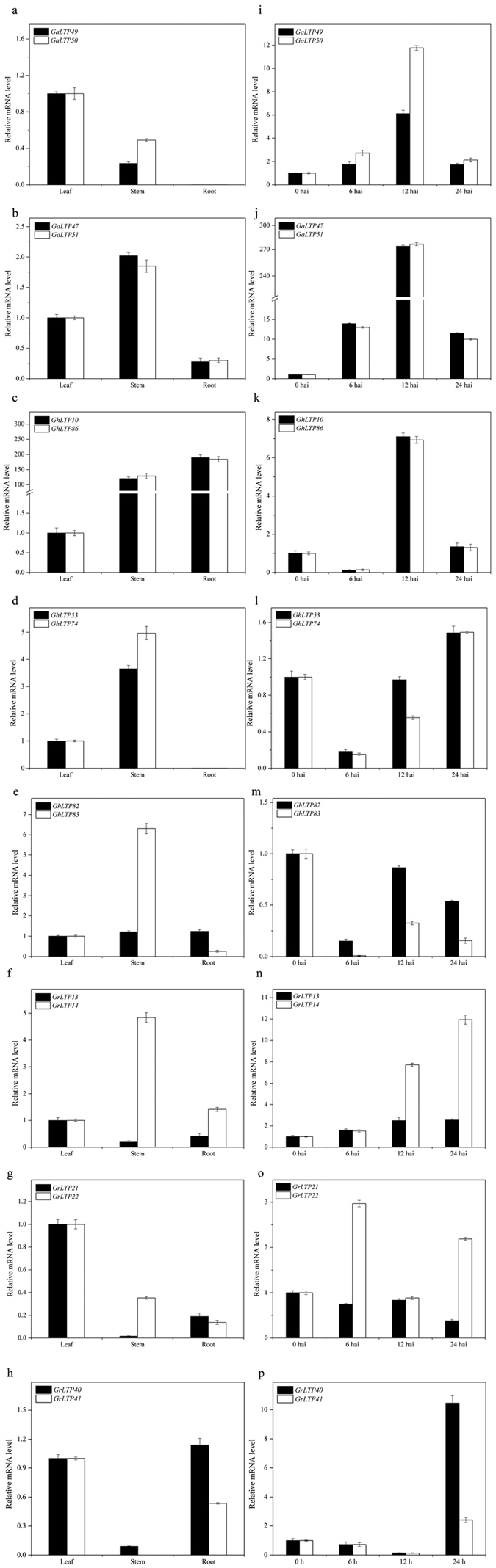
Quantitative RT-PCR analysis for selected duplicated *nsLTPs* from *G. arboreum, G. raimondii* and *G. hirsutum* in different organs (a–h) and leaves after *V. dahliae* treatment (i–p).

**Figure 7:**
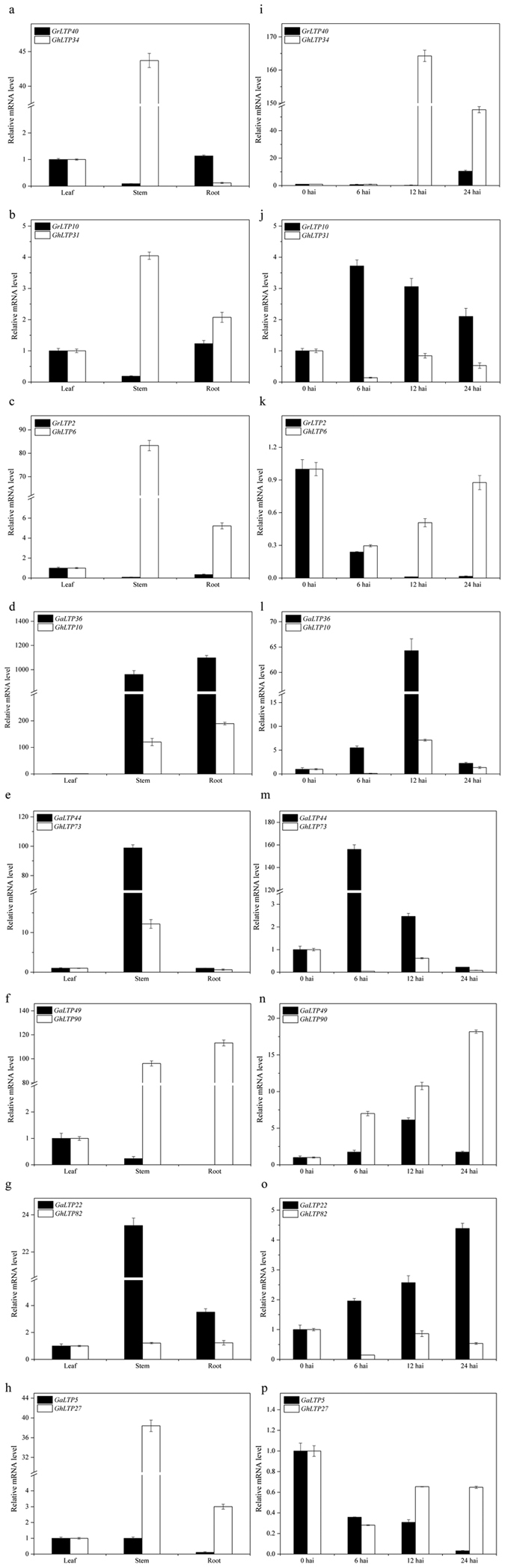
Expression patterns of the orthologous gene pairs from *G. arboreum, G. raimondii* and *G. hirsutum* in different organs (a–h) and leaves after *V. dahliae* treatment (i–p).

In the current study, expression patterns of the orthologous genes were compared in *G. raimondii, G. arboreum* and *G. hirsutum* (Figs 6 and 7 and Fig. S5). Among the intraspecific orthologous genes, 5 paired genes (*GaLTP47*/*51, GaLTP49*/*50, GhLTP10*/*86, GhLTP53*/*74* and *GrLTP40/41*) shared almost equivalent expression patterns in the tested tissues and leaves inoculated with *V. dahliae* (Fig. 6). However, this was not the case for *GhLTP82*/*83, GrLTP13*/*14* and *GrLTP21*/*22*. The expression patterns of these duplicated gene pairs were strongly divergent. *GhLTP83* and *GrLTP14* showed predominant expression in stems compared with *GhLTP82* and *GrLTP13*, respectively*. GrLTP21/22* also showed reciprocal silencing in roots and stems. Apart from the tissue-specific expression biases, *GhLTP82*/*83* and *GrLTP13*/*14* showed similar expression profiles after *V. dahliae* infection, while *GrLTP21*/*22* displayed reverse expression pattern in response to *V. dahliae*.

Among the interspecific orthologous genes, 10 pairs between *G. hirsutum* and *G. arboreum* and 6 pairs between *G. hirsutum* and *G. raimondii* were selected to evaluate their expression relationship (Fig. 7, Fig. S5). Among them, the 4 paired genes (*GaLTP25*/*GhLTP69, GaLTP35*/*GhLTP42, GaLTP36*/*GhLTP86* and *GaLTP36*/*GhLTP10*) shared similar expression patterns, and both genes were up-regulated after *V. dahliae* infection. However, the other 12 pairs displayed strong expression bias towards one copy in different organs, and the responses to *V. dahliae* were drastically different (Fig. 7, Fig. S5).

### Expression profile of *nsLTP* members in cotton

**Figure 8:**
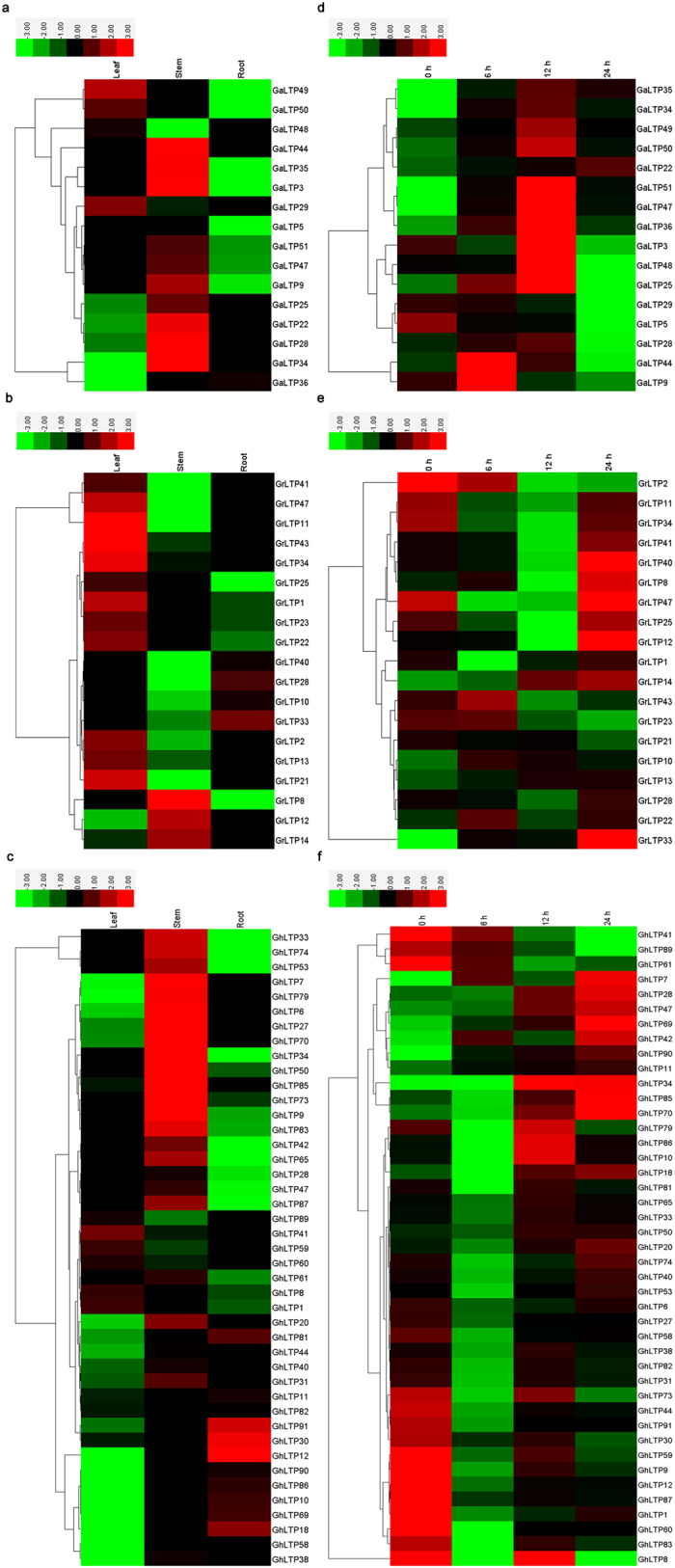
Heat map representation and hierarchical clustering of *GaLTPs, GrLTPs* and *GhLTPs* across different tissues (a–c) and in leaves after *V. dahliae* treatment (d–f). The color bar represents the relative signal intensity value.

In the current study, a total of 78 genes (16 *GaLTPs*, 19 *GrLTPs* and 43 *GhLTPs*) from all the subfamilies were selected, and the expression patterns of these genes varied significantly in different tissues (Fig. 8). Generally, Type V *nsLTPs* were mainly expressed at high levels in roots and young stems, while *nsLTPs* in Type I, II, IV and IX were predominantly expressed in stems and leaves. Additionally, *nsLTPs* play an important role in the protective mechanisms against pathogens in plants. The nsLTPs from some plants have reported to show obvious antifungal activities against *V. dahliae*^24^,^25^,^26^,^27^, we thus investigated the expression patterns of the *nsLTPs* in response to *V. dahliae* to take a further step towards understanding the function of cotton *nsLTPs* against *V. dahliae*. As displayed in Fig. 8, most *nsLTPs* in Type I and V showed a marked increase in transcript levels after *V. dahliae* inoculation, and different genes responded to different stages of *V. dahliae* infection. Some Type II *nsLTPs* were also slightly up-regulated in response to *V. dahliae* stress, while some *nsLTPs* in Type III, IV, VI, VIII and XI showed a decline in expression levels after treatment with *V. dahliae*.

## Discussion

The categorization of *nsLTPs* based on phylogenetic clustering provided comprehensive information about the gene family and facilitated further functional analysis. In the current study, the 189 *nsLTPs* identified in *G. raimondii, G. arboreum* and *G. hirsutum* were classified into 8 subfamilies (Type I, II, III, IV, V, VI, VIII and IX) through phylogenetic analysis, and no Type VII *nsLTPs* were found in *Gossypium* (Fig. 1, Fig. S1). It has been speculated that Type VII *nsLTPs* appeared specifically in monocots while Type IX *nsLTPs* were unique to dicots^3^,^9^, and our results in *Gossypium* further confirmed this viewpoint. In addition, *G. arboreum* lost all the Type III and Type IX *nsLTPs* (Fig. S2), suggesting that the evolution of plants not only involves gene retentions, but also is accompanied by gene losses and mutations^4^,^28^. Meanwhile, the proportion of *nsLTPs* in each subfamily indicated that Type I seemed to have contracted while Type VIII expanded in *G. hirsutum* compared with its diploid progenitors (Fig. S2b). The gene retentions and losses might be associated with the related functions during plant evolution^29^.

As for multigene families, the analysis of gene expression profiles often provides useful clues for functional assessment. The tissue-specific expression patterns of *nsLTPs* (Fig. 8) indicated their important roles in performing diverse developmental and physiological functions in cotton. Besides, Type I *nsLTPs* were reported to be involved in plant defense against phytopathogens^4^,^10^,^30^, and were classified as PR-14 family. Such a role is particularly consistent with our results (Fig. 8). Most Type I *nsLTPs* showed increased expression levels after *V. dahliae* infection. In addition, many Type V *nsLTPs* were also significantly induced after *V. dahliae* attack, suggesting that they may participate in the pathogen response. In a previous study, the expression of Type V *nsLTPs* was observed primarily in the vascular bundles, and they were deduced to be involved in defense signaling^10^. Additional work is needed to further characterize the role of Type V *nsLTPs* and their functional involvement in signal transduction. Moreover, the *nsLTPs* in other subgroups showed no expression changes or a decline of expression levels when treated with *V. dahliae*, suggesting that they may participate in other biological processes or respond to the attacks of other types of pathogens^3^,^4^,^30^,^31^,^32^.

Most nsLTPs within each subgroup shared common motif compositions, and the motif distributions among the different cotton species did not vary much (Fig. 3). Motif 5 and 7 corresponded to the highly conserved residues located in Pro-Tyr-X-Ile-Ser and Thr/Ser-X1-X2-Asp-Arg/Lys, respectively, and the two consensus pentapeptides were only found in Type I nsLTPs. Our result is consistent with previous studies^4^,^33^. Besides, the properties of the amino acid may determine the Cys pairing style, thus influencing the overall folding of the protein^30^,^32^,^34^. Generally, nsLTP1 has a hydrophilic amino acid in the CXC motif, whereas nsLTP2 contains a hydrophobic residue in the same position^30^,^32^. In our study, Motif 10 represented the pattern with a hydrophilic residue separating Cys_5_ and Cys_6_ in Type I, while Motif 1, 2, 6 and 15 corresponded to the Cys_5_XCys_6_ with a hydrophobic residue in the central position in the other subfamilies. Among the five hydrophobic residues in the CXC motif, Leu is the most frequent residue (75.40%), and this result is in accordance with previous studies^3^,^4^. Besides, Cys_5_-Val-Cys_6_, which corresponded to Motif 15, was only found in four Type VI nsLTPs.

The intron-exon pattern carries the imprint of the evolution of a gene family^35^,^36^. Previous studies have indicated the generality that some *nsLTP*s in Type I, III, IV, V and VI contained introns, while no introns were identified in Type II, VIII and IX *nsLTPs*^3^,^4^,^32^. However, our result showed some differences. In *Gossypium*, introns were found in Type VIII and IX, while no introns were identified in Type III (Fig. 3). The *nsLTP* family evolved in early diverged land plants, and new subfamilies evolved during land plant evolution^2^,^32^. Intron loss events were considered the main cause for the formation of novel *nsLTP* types, and also contributed to novel gene formation within the specific gene subfamilies^32^. Therefore, compared with the *nsLTPs* in other plants, Type IX and some Type VIII *nsLTPs* in *Gossypium* still possessed introns, while Type III genes have evolved with no introns harbored. The adoption of different *nsLTP* types could help evolved plants adapt to the stressful conditions on land gradually, so the *nsLTP* family was selected and expanded in land plants^2^,^32^.

The role of gene duplication in the genesis of evolutionary novelty and complexity has long been recognized^37^,^38^. The results in our study indicated that both tandem and segmental duplication events contributed to the expansion of the *nsLTP* family in *G. arboreum* and *G. hirsutum*, while tandem duplication contributed to the *nsLTP* expansion in *G. raimondii* (Fig. 4, Table 2). Previous studies have indicated that the ancient and recent whole-genome duplication (WGD) events occurred in *Gossypium* approximately 115–146 and 13–20 MYA, respectively, and the predicted hybridization and formation of allotetraploid cotton occurred approximately 1.5 MYA^14^,^15^,^23^. In the current study, most of the *nsLTP* duplication events in *G. arboreum* and *G. raimondii* might have occurred fewer than 32.69 MYA, and were considered the recent duplication events. The duplication event occurred 194.74 MYA in *G. arboreum* was therefore regarded as the ancient hexaploidization event. In *G. hirsutum*, the duplication events occurred approximately 5.84–24.50 MYA, suggesting that these duplication events might have occurred before the interspecific hybridization and subsequent polyploidization in *G. hirsutum*.

In addition, the orthologous genes in *G. arboreum, G. raimondii* and *G. hirsutum* were identified (Fig. 5, Table S4). The results revealed that 31 *GaLTPs* lost their orthologs in the A_t_ subgenome of *G. hirsutum* and 24 *GrLTPs* lost their orthologs in the D_t_ subgenome. These genes were mainly distributed in Type I, Type II, Type IV and Type V, and the proportions of these *GrLTP* and *GaLTP* genes distributed in each subfamily were complementary (Fig. S3). For example, in Type I, 66.67% of the *GaLTPs* had no orthologs in the A_t_ subgenome, whereas a relatively lower ratio (50%) of the *GrLTPs* lost their orthologs in the D_t_ subgenome. On the contrary, in Type V, only 33.33% of the *GaLTPs* lost their orthologs, while many more *GrLTPs* (75%) lost their orthologs. The ratios in other subfamilies such as Type IV, VI and VIII showed similar complementary phenomena, suggesting that the loss and retention of *nsLTPs* in *G. hirsutum* might not be random. During the process of polyploidization, chromosomal recombinations and rearrangements resulted in gene losses and gene retentions as duplicate orthologs^39^,^40^. The regular losses in conjunction with retentions might have avoided severe functional loss and redundancy of the genes in plants.

Duplications of genomic content often occur during DNA replication and recombination through independent mechanisms^38^,^41^. Some duplicated genes might have been subjected to diversification of their functions due to the divergent changes in their protein sequences together with *cis*-regulatory elements^37^. The *nsLTPs* may have undergone independent duplication events followed by functional diversification in each species during speciation^42^,^43^. In rice and wheat, the *nsLTPs* may have suffered from a complex evolutionary selection mechanism including subfunctionalization^43^,^44^. The functional diversification of *nsLTPs* appears to be not random, but seems to have originated from the specific biological processes over the course of evolution. In the current study, five pairs of duplicated genes shared similar expression profiles (Fig. 6). These results indicated that these duplicated genes might retain some essential functions during subsequent evolution, and the similar expression patterns may be related to the highly similar protein architecture of these duplicated genes. However, three paired duplicated genes showed significant expression divergence (Fig. 6). The diversity might be caused by the significant variation in gene regulation after the duplication events^45^,^46^. The differential expression patterns of duplicated genes in *Gossypium* demonstrated that these genes might have experienced functionalization during the evolutionary process.

As a prominent mode of speciation in flowering plants, allopolyploidy can cause genomic changes, including chromosomal rearrangements and changes in gene expression^45^,^46^,^47^,^48^. Recent studies have indicated the unequal expression of orthologous genes in allotetraploids such as *Gossypium*^13^,^49^, *Brassica*^47^, *Oryza minuta*^50^ and the synthetic allopolyploid Arabidopsis^51^, and the gene expression levels in these allopolyploids deviated from the expression of their orthologous genes in the progenitors. In our study, the expression patterns of genes in *G. hirsutum* revealed considerable differences from the expression of their orthologous genes in its progenitors (Fig. 7 and Fig. S5), indicating that the functions of the orthologous genes were strongly differentiated over the course of evolution.

In summary, 189 *nsLTPs* were identified in *G. raimondii, G. arboreum* and *G. hirsutum* in this study. Comprehensive study of the *nsLTPs* in *Gossypium* provided some important features of the gene family such as gene structure, expansion and expression. The findings here provide researchers with novel findings about the molecular evolution and expansion history of the *nsLTP* family in *Gossypium*, and offer a good opportunity to further investigate the *nsLTP* family in plants, especially polyploid plants. Besides, the analysis of the cotton *nsLTPs* in response to *V. dahliae* might help guide future identification of disease-resistant genes and exploitation of wilt-resistant varieties.

## Materials and Methods

### Plant materials and *V. dahliae* spore treatment

The seeds of *G. raimondii, G. arboreum* (Shixiya1) and *G. hirsutum* (TM-1) were kindly supplied by National Medium-term Gene Bank of Cotton in China. Cotton seeds were surface-sterilized with 0.1% HgCl_2_ for 10–15 min and washed with sterile water for at least 5 times. Sterilized seeds were sown on the half-strength Murashige and Skoog (MS) solid medium. After 3-day germination, the seedlings were transferred to half-strength Hoagland nutrient solution at pH 6.0. Fresh nutrient solution was continuously aerated with pumps and air stones, and was changed every 3 days. Plants were cultivated in a growth chamber at 28 °C with a photoperiod of 16 h light and 8 h dark. For tissue-specific expression analysis, roots, stems and leaves were harvested from 3-week-old cotton seedlings, and quick-frozen in liquid nitrogen, then stored at −80 °C until RNA isolation. Three plants constituted an individual replicate and three biological replicates were sampled for RNA extraction.

*V. dahliae* isolate Anyang (ACCC no. 36207) is a popular *V. dahliae* strain isolated from Anyang, Henan Province, China. It was supplied by the Cotton Research Institute of the Chinese Academy of Agricultural Science and preserved at the Agricultural Culture Collection, Beijing, China. *V. dahliae* mycelia growing on potato dextrose agar (PDA) plates were transferred to Czapek’s medium (containing 2.0 g·l^−1^ NaNO_3_, 1.0 g·l^−1^ K_2_HPO_4_, 0.5 g·l^−1^ MgSO_4_ •7H_2_O, 1.0 g·l^−1^ KCl, 0.01 g·l^−1^ FeSO_4_•7H_2_O and 30.0 g·l^−1^ sucrose) on a shaker at 150 rpm at 25 °C for sub-culturing. The spore concentration was determined with a haemocytometer and adjusted to a concentration of 10^7^ spores ml^−1^ with sterile distilled water prior to use. Three-week old plants were inoculated with *V. dahliae* spores using the root-dip method, and the leaves were collected 0 h, 6 h, 12 h and 24 h after *V. dahliae* inoculation to examine the expression patterns of the *nsLTP* family in cotton under *V. dahliae* treatment.

### RNA isolation and quantitative real-time PCR (qRT-PCR)

Total RNA was extracted using an RNAprep Pure Plant kit (Tiandz, China), and first-strand cDNA was synthesized from DNase-treated RNA with a PrimerScript 1st Strand cDNA synthesis kit (TaKaRa). Gene-specific primers were designed based on their coding sequences (CDSs) and then synthesized commercially (Generay, Shanghai, China) (Table S5). qRT-PCR was performed with SYBR premix Ex Taq (TaKaRa) and the CFX96 Realtime System (Bio-Rad, France) by strictly following the manufacturer’s instructions. The qRT-PCR machine was programmed with 40 cycles and an annealing temperature of 60 °C. The cotton *EF1α* gene was used as an endogenous control for all the qRT-PCR analyses. The relative transcription levels were calculated using the 2^−ΔΔCT^ method. Three technical replicates were performed for each sample. The expression profiles of the *nsLTPs* were clustered using Cluster 3.0 software^52^.

### Sequence retrieval and structural analysis

All the nsLTP sequences of Arabidopsis (Table S1) were collected from TAIR (http://www.arabidopsis.org/index.jsp) and were used as queries by searching against the cotton genome database^14^,^15^,^23^ using the BLASTP program with default parameters (E_value ≤10) (http://cgp.genomics.org.cn/page/species/index.jsp). The conserved LTP domain of each putative LTP sequence in *Gossypium* was confirmed in the Conserved Domain Database in National Center for Biotechnology Information (NCBI) (http://www.ncbi.nlm.nih.gov). Then, the candidate nsLTP sequences were manually examined for the presence of the 8 CM, and proteins lacking the essential Cys residues were excluded. After that, the putative proline-rich proteins and the proteins without NSSs (checked with SignalP 4.0, http://www.cbs.dtu.dk/services/SignalP) were also removed. Additionally, the proteins with C-terminal GPI anchor signals (predicted with big-PI Plant Predictor, PSORT and PredGPI) were excluded^53^,^54^,^55^. The protein sequences of the 2S- albumins (At2S1 to At2S4)^56^ and alpha amylase inhibitor (RATI)^57^ were then BLAST-searched against the rest of the candidate nsLTPs to exclude possible inhibitors and cereal storage proteins. Moreover, the proteins with more than 120 amino acids at maturity were discarded. Through these searching and screening procedures, the rest of the nsLTPs were finally confirmed and used for the following analysis. Primary and secondary protein structures were predicted with ProtParam (http://web.expasy.org/protparam/) and SOPMA (http://npsa-pbil.ibcp.fr/cgi-bin/npsa_automat.pl?page=npsa_sopma.html). In addition, the nsLTPs were clustered using OrthoMCL to identify the orthologous groups (OGs)^58^. The inflation parameter was set to the default.

### Phylogenetic analysis and sequence alignment

All the nsLTP sequences in this study were aligned using ClustalX version 2.1^59^. The PHYLIP program (http://evolution.genetics.washington.edu/phylip.html) was used to estimate the maximum-likelihood phylogeny for all the nsLTP sequences in Arabidopsis and the three *Gossypium* species with the JTT model. In addition, MrBayes version 3.1.2 was used to conduct Bayesian analysis for the nsLTP sequences in the three *Gossypium* species^60^. Trees were visualized with Figtree version 1.4.0 (http://tree.bio.ed.ac.uk/software/figtree/).

### Protein motif and gene structure analysis

The conserved motifs of the nsLTPs were investigated using the online MEME program^61^. The analysis was performed with a set of parameters as follows: the optimum motif width was set to ≥6 and ≤200; the maximum number of motifs was set to 20^62^. Sequence logos for the conserved domains were generated using the WebLogo tool (http://weblogo.berkeley.edu/). Genomic schematic diagrams of the *nsLTPs* were obtained by comparing the genomic sequences and their predicted CDSs using the GSDS tool (http://gsds.cbi.pku.edu.cn/).

### Chromosomal mapping and gene duplications

The chromosome location information of the *nsLTPs* was searched in the cotton genome database. MapInspect software (http://www.plantbreeding.wur.nl/uk/software-mapinspect.html) was used to generate chromosomal distribution images for these *nsLTPs* in *G. raimondii, G. arboreum* and *G. hirsutum*. Gene duplication events were investigated as described in previous reports^62^. Orthologous groups of the *nsLTP* family among the three *Gossypium* species were generated using the OrthoMCL database. Conserved syntenic blocks were inferred by running the OrthoClusterDB tool available at GDR (http://genome.sfu.ca/cgi-bin/orthoclusterdb/runortho.cgi). The diagram was plotted using Circos^63^. Furthermore, the selection pressure for each orthologous gene pair was calculated by the Ka (non-synonymous substitution rate)/Ks (synonymous substitution rate) ratio^64^. The formula “t = Ks/2r” was used to estimate the divergence time, and a neutral substitution rate (r) of 2.6 × 10^−9^ was used in the current study.

## Additional Information

**How to cite this article:** Li, F. *et al*. Genomic Identification and Comparative Expansion Analysis of the *Non-Specific Lipid Transfer Protein* Gene Family in *Gossypium. Sci. Rep.* **6**, 38948; doi: 10.1038/srep38948 (2016).

**Publisher's note:** Springer Nature remains neutral with regard to jurisdictional claims in published maps and institutional affiliations.

## Supplementary Material

Supplementary Information

## Figures and Tables

**Table 1:** Diversity of eight cysteine motifs in different types of nsLTPs identified in *Gossypium.*

Type	Number of members	Spacing pattern												
Type I	63	X_2-5,7,9_	C	X_9_	C	X_13-16_	CC	X_19,20_	CXC	X_19,21-24_	C	X_13-15_	C	X_2-5,7,8,10,19-21,23,25_
Type II	44	X_2,5,6,8-11,13,14_	C	X_7,8_	C	X_12-14_	CC	X_8_	CXC	X_23_	C	X_6,7,9_	C	X_0_
Type III	4	X_2,8_	C	X_9_	C	X_16_	CC	X_9_	CXC	X_12_	C	X_6_	C	X_1,2,4_
Type IV	22	X_3,6_	C	X_9_	C	X_15,17_	CC	X_9_	CXC	X_22,24_	C	X_7,9_	C	X_0,2,7,11,12_
Type V	32	X_3,7_	C	X_14_	C	X_14_	CC	X_11,12_	CXC	X_24_	C	X_10_	C	X_3,4,6,7,12,14,17,19,23_
Type VI	7	X_1,5,6,9_	C	X_10_	C	X_12,16_	CC	X_9_	CXC	X_22_	C	X_9_	C	X_6,8-11_
Type VIII	11	X_3,4,8_	C	X_6_	C	X_14_	CC	X_12_	CXC	X_25,27_	C	X_8_	C	X_6,13,16,20,21,24,37_
Type IX	3	X_2_	C	X_13_	C	X_15_	CC	X_9_	CXC	X_22_	C	X_6_	C	X_4_

Character “X” represents any amino acid, and the Arabic numeral following “X” stands for the number of amino acid residues.

**Table 2:** Ka/Ks analysis for the duplicated gene pairs.

Species	Duplicated gene 1	Duplicated gene 2	Subfamily	Ka	Ks	Ka/Ks	Purifing selection	Duplicate type	Age (MYA)
*G. arboreum*	*GaLTP12*	*GaLTP45*	Type II	2.51E-07	5.85E-07	0.42952	Yes	segmental	0.00011255
	*GaLTP18*	*GaLTP20*	Type V	0.00737363	0.165432	0.0445719	Yes	tandem	31.8138462
	*GaLTP37*	*GaLTP38*	Type I	0.0728347	0.160261	0.454475	Yes	tandem	30.8194231
	*GaLTP39*	*GaLTP42*	Type II	0.0312662	0.0690421	0.452857	Yes	tandem	13.2773269
	*GaLTP40*	*GaLTP41*	Type II	0.0261638	0.111607	0.234428	Yes	tandem	21.4628846
	*GaLTP47*	*GaLTP51*	Type V	0.995287	1.01263	0.982869	Yes	segmental	194.736538
	*GaLTP49*	*GaLTP50*	Type I	0.0358127	0.0426301	0.84008	Yes	tandem	8.19809615
	*GrLTP13*	*GrLTP14*	Type II	0.0254554	0.128337	0.198347	Yes	tandem	24.6801923
	*GrLTP21*	*GrLTP22*	Type V	0.0185999	0.164891	0.112801	Yes	tandem	31.7098077
*G. raimondii*	*GrLTP26*	*GrLTP27*	Type I	2.48E-07	6.24E-07	0.397702	Yes	tandem	0.00011993
	*GrLTP40*	*GrLTP41*	Type I	0.0493708	0.0199624	2.47319	No	tandem	3.83892308
	*GrLTP45*	*GrLTP46*	Type II	0.00500846	0.000100169	50	No	tandem	0.01926327
	*GhLTP10*	*GhLTP86*	Type V	0.0168826	0.0310859	0.543095	Yes	segmental	5.97805769
	*GhLTP22*	*GhLTP23*	Type II	0.0148626	0.0889965	0.167002	Yes	tandem	17.1147115
*G. hirsutum*	*GhLTP51*	*GhLTP52*	Type II	0.0187175	0.0303504	0.616713	Yes	tandem	5.83661538
	*GhLTP53*	*GhLTP74*	Type II	0.0663427	0.0389369	1.70385	No	segmental	7.48786538
	*GhLTP76*	*GhLTP77*	Type IV	0.066261	0.127397	0.520114	Yes	tandem	24.4994231
	*GhLTP82*	*GhLTP83*	Type VIII	0.0595669	0.102935	0.578685	Yes	tandem	19.7951923

## References

[b1] Kader J. Lipid-transfer proteins in plants. Annu. Rev. Plant Biol. 47, 627–654 (1996). 10.1146/annurev.arplant.47.1.627 15012303

[b2] Liu F. . Non-specific lipid transfer proteins in plants: presenting new advances and an integrated functional analysis. J. Exp. Bot. 10.1093/jxb/erv313 (2015). 26139823

[b3] Boutrot F., Chantret N. & Gautier M. Genome-wide analysis of the rice and Arabidopsis n*on-specific lipid transfer protein* (*nsLtp*) gene families and identification of wheat *nsLtp* genes by EST data mining. BMC Genomics 9, 86, 10.1186/1471-2164-9-86 (2008). 18291034 PMC2277411

[b4] Li J. . Genome-wide survey and expression analysis of the putative non-specific lipid transfer proteins in *Brassica rapa* L. PLoS ONE 9, e84556, 10.1371/journal.pone.0084556 (2014). 24497919 PMC3908880

[b5] Sels J., Mathys J., De Coninck B. M., Cammue B. P. & De Bolle M. F. Plant pathogenesis-related (PR) proteins: a focus on PR peptides. Plant Physiol. Bioch. 46, 941–950 (2008). 10.1016/j.plaphy.2008.06.011 18674922

[b6] Regente M. C., Giudici A. M., Villalain J. & Canal L. The cytotoxic properties of a plant lipid transfer protein involve membrane permeabilization of target cells. Lett. Appl. Microbiol. 40, 183–189 (2005). 15715642 10.1111/j.1472-765X.2004.01647.x

[b7] Zhu X. . Overexpression of wheat lipid transfer protein gene *TaLTP5* increases resistances to *Cochliobolus sativus* and *Fusarium graminearum* in transgenic wheat. Funct. Integr. Genomic 12, 481–488 (2012). 10.1007/s10142-012-0286-z 22689341

[b8] Jung H. W., Kim W. & Hwang B. K. Three pathogen-inducible genes encoding lipid transfer protein from pepper are differentially activated by pathogens, abiotic, and environmental stresses. Plant, cell & environment 26, 915–928 (2003). 10.1046/j.1365-3040.2003.01024.x 12803619

[b9] Liu W. . Discovery, identification and comparative analysis of non-specific lipid transfer protein (nsLtp) family in *Solanaceae. Genomics, proteomics &* bioinformatics 8, 229–237 (2010). 10.1016/S1672-0229(10)60024-1 PMC5054125 21382591

[b10] Wang H. W. . Insight into the molecular evolution of non-specific lipid transfer proteins via comparative analysis between rice and sorghum. DNA Res. 10.1093/dnares/dss003 (2012). PMC3325081 22368182

[b11] Wang N. . Construction and analysis of a plant non-specific lipid transfer protein database (nsLTPDB). BMC Genomics 13, 1, 10.1186/1471-2164-13-S1-S9 (2012). 22369214 PMC3303721

[b12] Tapia G., Morales-Quintana L., Parra C., Berbel A. & Alcorta M. Study of nsLTPs in *Lotus japonicus* genome reveal a specific epidermal cell member (LjLTP10) regulated by drought stress in aerial organs with a putative role in cutin formation. Plant Mol. Biol. 82, 485–501 (2013). 23733601 10.1007/s11103-013-0080-x

[b13] Flagel L. E., Wendel J. F. & Udall J. A. Duplicate gene evolution, homoeologous recombination, and transcriptome characterization in allopolyploid cotton. BMC Genomics 13, 302, 10.1186/1471-2164-13-302 (2012). 22768919 PMC3427041

[b14] Li F. . Genome sequence of the cultivated cotton *Gossypium arboreum*. Nat. Genet. 46, 567–572 (2014). 24836287 10.1038/ng.2987

[b15] Li F. . Genome sequence of cultivated Upland cotton (*Gossypium hirsutum* TM-1) provides insights into genome evolution. Nat. Biotechnol. 33, 524–530 (2015). 25893780 10.1038/nbt.3208

[b16] Wendel J. F., Schnabel A. & Seelanan T. Bidirectional interlocus concerted evolution following allopolyploid speciation in cotton (*Gossypium*). P. Nat. Acad. Sci. 92, 280–284 (1995). 10.1073/pnas.92.1.280 PMC42862 7816833

[b17] Paterson A. H. . Repeated polyploidization of *Gossypium* genomes and the evolution of spinnable cotton fibres. Nature 492, 423–427 (2012). 23257886 10.1038/nature11798

[b18] Zhang W. W. . Cotton gene expression profiles in resistant *Gossypium hirsutum* cv. Zhongzhimian KV1 responding to *Verticillium dahliae* strain V991 infection. Mol. Biol. Rep. 39, 9765–9774 (2012). 22733494 10.1007/s11033-012-1842-2

[b19] Zhao Y., Wang H., Chen W. & Li Y. Genetic structure, linkage disequilibrium and association mapping of *Verticillium* wilt resistance in elite cotton (*Gossypium hirsutum* L.) germplasm population. PLoS ONE 9, e86308, 10.1371/journal.pone.0086308 (2014). 24466016 PMC3900507

[b20] Chen J. . Genome-wide analysis of the gene families of resistance gene analogues in cotton and their response to *Verticillium* wilt. BMC Plant Biol. 15, 148, 10.1186/s12870-015-0508-3 (2015). 26084488 PMC4471920

[b21] Feng J. . Analysis of five differentially expressed gene families in fast elongating cotton fiber. Acta Bioch. Bioph. Sin. 36, 51–56 (2004). 10.1093/abbs/36.1.51 14732876

[b22] Gao P. . Identification of genes preferentially expressed in cotton fibers: A possible role of calcium signaling in cotton fiber elongation. Plant Sci. 173, 61–69 (2007).

[b23] Wang K. . The draft genome of a diploid cotton *Gossypium raimondii*. Nat Genet. 44, 1098–1103 (2012). 22922876 10.1038/ng.2371

[b24] Cammue B. P. A. . A potent antimicrobial protein from onion seeds showing sequence homology to plant lipid transfer proteins. Plant Physiol. 109, 445–455 (1995). 7480341 10.1104/pp.109.2.445 PMC157606

[b25] Yili A. . Fungicidal lipid-transfer peptide from *Daucus carota sativa* seeds. Chem. Nat. Compd. 43, 450–453 (2007).

[b26] Yang X., Xiao Y., Wang X. & Pei Y. Expression of a novel small antimicrobial protein from the seeds of motherwort (*Leonurus japonicus*) confers disease resistance in tobacco. Appl. Environ. Microb. 73, 939–946 (2007). 10.1128/AEM.02016-06 PMC1800757 17158620

[b27] Sun J. Y. . Characterization and antifungal properties of wheat nonspecific lipid transfer proteins. Mol. Plant Microbe In. 21, 346–360 (2008). 10.1094/MPMI-21-3-0346 18257684

[b28] Krishnamurthy P., Kim J. A., Jeong M., Kang C. H. & Lee S. I. Defining the RNA-binding glycine-rich (RBG) gene superfamily: new insights into nomenclature, phylogeny and evolutionary trends obtained by genome-wide comparative analysis of Arabidopsis, Chinese cabbage, rice and maize genomes. Mol. Genet. Genomics 290, 2279–2295 (2015). 26123085 10.1007/s00438-015-1080-0

[b29] Fan K. . Molecular evolution and expansion analysis of the NAC transcription factor in *Zea mays*. PLoS ONE 9, e111837, 10.1371/journal.pone.0111837 (2014). 25369196 PMC4219692

[b30] Wei K. & Zhong X. Non-specific lipid transfer proteins in maize. BMC Plant Biol. 14, 281, 10.1186/s12870-014-0281-8 (2014). 25348423 PMC4226865

[b31] Cotta M. G. . Lipid transfer proteins in coffee: isolation of *Coffea* orthologs, *Coffea arabica* homeologs, expression during coffee fruit development and promoter analysis in transgenic tobacco plants. Plant Mol. Biol. 85, 11–31 (2014). 24469961 10.1007/s11103-013-0166-5

[b32] Edstam M. M., Viitanen L., Salminen T. A. & Edqvist J. Evolutionary history of the non-specific lipid transfer proteins. Mol. Plant 4, 947–964 (2011). 21486996 10.1093/mp/ssr019

[b33] Douliez J., Michon T., Elmorjani K. & Marion D. Mini review: structure, biological and technological functions of lipid transfer proteins and indolines, the major lipid binding proteins from cereal kernels. J. Cereal. Sci. 32, 1–20 (2000).

[b34] Douliez J. P., Pato C., Rabesona H., Mollé D. & Marion D. Disulfide bond assignment, lipid transfer activity and secondary structure of a 7-kDa plant lipid transfer protein, LTP2. Eur. J. Biochem. 268, 1400–1403 (2001). 11231292 10.1046/j.1432-1327.2001.02007.x

[b35] Lynch M. Intron evolution as a population-genetic process. P. Nat. Acad. Sci. 99, 6118–6123 (2002). 10.1073/pnas.092595699 PMC122912 11983904

[b36] Del Campo E. M., Casano L. M. & Barreno E. Evolutionary implications of intron-exon distribution and the properties and sequences of the *RPL10A* gene in eukaryotes. Mol. Phylogenet. Evol. 66, 857–867 (2013). 23201395 10.1016/j.ympev.2012.11.013

[b37] Moore R. C. & Purugganan M. D. The evolutionary dynamics of plant duplicate genes. Curr. Opin. Plant Biol. 8, 122–128 (2005). 15752990 10.1016/j.pbi.2004.12.001

[b38] Flagel L. E. & Wendel J. F. Gene duplication and evolutionary novelty in plants. New Phytol. 183, 557–564 (2009). 19555435 10.1111/j.1469-8137.2009.02923.x

[b39] Paterson A. H. . Many gene and domain families have convergent fates following independent whole-genome duplication events in *Arabidopsis, Oryza, Saccharomyces* and *Tetraodon*. Trends Genet. 22, 597–602 (2006). 16979781 10.1016/j.tig.2006.09.003

[b40] Buggs R. J. . Rapid, repeated, and clustered loss of duplicate genes in allopolyploid plant populations of independent origin. Curr. Biol. 22, 248–252 (2012). 22264605 10.1016/j.cub.2011.12.027

[b41] Ramsey J. & Schemske D. W. Pathways, mechanisms, and rates of polyploid formation in flowering plants. Annu. Rev. Eco. Syst. 29, 467–501 (1998).

[b42] Jang C. S. . Divergence of genes encoding non-specific lipid transfer proteins in the *Poaceae* family. Mol. Cells 24, 215–223 (2007). 17978574

[b43] Jang C. S. . Evolution of *non-specific lipid transfer protein* (*nsLTP*) genes in the *Poaceae* family: their duplication and diversity. Mol. Genet. Genomics 279, 481–497 (2008). 18270740 10.1007/s00438-008-0327-4

[b44] Wang H. W. . Expressional diversity of wheat *nsLTP* genes: evidence of subfunctionalization via *cis*-regulatory divergence. Genetica 138, 843–852 (2010). 20532958 10.1007/s10709-010-9467-7

[b45] Adams K. L. Evolution of duplicate gene expression in polyploid and hybrid plants. J. Hered. 98, 136–141 (2007). 17208934 10.1093/jhered/esl061

[b46] Wang J., Tian L., Lee H. & Chen Z. J. Nonadditive regulation of *FRI* and *FLC* loci mediates flowering-time variation in Arabidopsis allopolyploids. Genetics 173, 965–974 (2006). 16547097 10.1534/genetics.106.056580 PMC1526503

[b47] Zhang X. . Interspecific hybridization, polyploidization, and backcross of *Brassica oleracea* var. *alboglabra* with *B. rapa* var. *purpurea* morphologically recapitulate the evolution of *Brassica* vegetables. Sci. Rep. 6, 18618, 10.1038/srep18618 (2016). 26727246 PMC4698638

[b48] Wang J. . Genomewide nonadditive gene regulation in Arabidopsis allotetraploids. Genetics 172, 507–517 (2006). 16172500 10.1534/genetics.105.047894 PMC1456178

[b49] Yoo M. J., Szadkowski E. & Wendel J. F. Homoeolog expression bias and expression level dominance in allopolyploid cotton. Heredity 110, 171–180 (2013). 23169565 10.1038/hdy.2012.94 PMC3554454

[b50] Sui Y., Li B., Shi J. & Chen M. Genomic, regulatory and epigenetic mechanisms underlying duplicated gene evolution in the natural allotetraploid *Oryza minuta*. BMC Genomics 15, 1, 10.1186/1471-2164-15-11 (2014). 24393121 PMC3890553

[b51] Akama S., Shimizu-Inatsugi R., Shimizu K. K. & Sese J. Genome-wide quantification of homeolog expression ratio revealed nonstochastic gene regulation in synthetic allopolyploid Arabidopsis. Nucleic Acids Res. 42, e46, 10.1093/nar/gkt1376 (2014). 24423873 PMC3973336

[b52] de Hoon M. J., Imoto S., Nolan J. & Miyano S. Open source clustering software. Bioinformatics 20, 1453–1454 (2004). 14871861 10.1093/bioinformatics/bth078

[b53] PSORT I. I. PSORT: a program for detecting sorting signals in proteins and predicting their subcellular localization. J. Mol. Biol. 266, 594–600 (1997). 10087920 10.1016/s0968-0004(98)01336-x

[b54] Pierleoni A., Martelli P. L. & Casadio R. PredGPI: a GPI-anchor predictor. BMC Bioinformatics 9, 392, 10.1186/1471-2105-9-392 (2008). 18811934 PMC2571997

[b55] Eisenhaber B. . Glycosylphosphatidylinositol lipid anchoring of plant proteins. Sensitive prediction from sequence-and genome-wide studies for Arabidopsis and rice. Plant Physiol. 133, 1691–1701 (2003). 14681532 10.1104/pp.103.023580 PMC300724

[b56] Guerche P. . Differential expression of the Arabidopsis *2S albumin* genes and the effect of increasing gene family size. Plant Cell 2, 469–478 (1990). 12354963 10.1105/tpc.2.5.469 PMC159903

[b57] Alam N., Gourinath S., Dey S., Srinivasan A. & Singh T. P. Substrate-inhibitor interactions in the kinetics of α-amylase inhibition by ragi α-amylase/trypsin inhibitor (RATI) and its various N-terminal fragments. Biochemistry-US 40, 4229–4233 (2001). 10.1021/bi002537v 11284678

[b58] Li L., Stoeckert C. J. & Roos D. S. OrthoMCL: identification of ortholog groups for eukaryotic genomes. Genome Res. 13, 2178–2189 (2003). 12952885 10.1101/gr.1224503 PMC403725

[b59] Larkin M. A. . Clustal W and Clustal X version 2.0. Bioinformatics 23, 2947–2948 (2007). 17846036 10.1093/bioinformatics/btm404

[b60] Huelsenbeck J. P. & Ronquist F. MRBAYES: Bayesian inference of phylogenetic trees. Bioinformatics 17, 754–755 (2001). 11524383 10.1093/bioinformatics/17.8.754

[b61] Bailey T. L., Johnson J., Grant C. E. & Noble W. S. The MEME Suite. Nucleic Acids Res. 43, W39–W49 (2015). 25953851 10.1093/nar/gkv416 PMC4489269

[b62] Zhao Y. . Systematic analysis of sequences and expression patterns of drought-responsive members of the *HD-Zip* gene family in maize. PLoS ONE 6, e28488, 10.1371/journal.pone.0028488 (2011). 22164299 PMC3229603

[b63] Krzywinski M. . Circos: an information aesthetic for comparative genomics. Genome Res. 19, 1639–1645 (2009). 19541911 10.1101/gr.092759.109 PMC2752132

[b64] Zhang Z. . KaKs_Calculator: calculating Ka and Ks through model selection and model averaging. Genomics, proteomics & bioinformatics 4, 259–263 (2006). 10.1016/S1672-0229(07)60007-2 PMC5054075 17531802

