# Supplementary material for: Genomic Identification and Comparative Expansion Analysis of the Non-Specific Lipid Transfer Protein Gene Family in Gossypium
Source: Sci Rep. 2016 Dec 15;6:38948. doi: 10.1038/srep38948 (PMC5157027; doi:10.1038/srep38948)
Supplement: Supplementary Information [file srep38948-s1.pdf]

Electronic Supplementary Information

The title: Genomic Identification and Comparative Expansion Analysis of the *Non-Specific Lipid Transfer Protein* Gene Family in *Gossypium*

The journal: Scientific Reports

The names of the authors: Feng Li[1†], Kai Fan[1,2†], Fanglu Ma[1], Erkui Yue[1], Noreen Bibi[1,3], Ming Wang[1], Hao Shen[1], Md Mosfeq-Ul Hason[1], Xuede Wang[1*]

The affiliation and address of the authors are as follows:

[1]Institute of Crop Science, College of Agriculture and Biotechnology, Zhejiang University, Hangzhou 310058, People's Republic of China

[2]College of Crop Science, Fujian Agriculture and Forestry University, Fuzhou, 350002, Fujian, China

[3]Nuclear Institute for Agriculture and Biology, Faisalabad, Pakistan

[*]Corresponding author. Email: xdwang@zju.edu.cn

[†]These authors contributed equally to this work

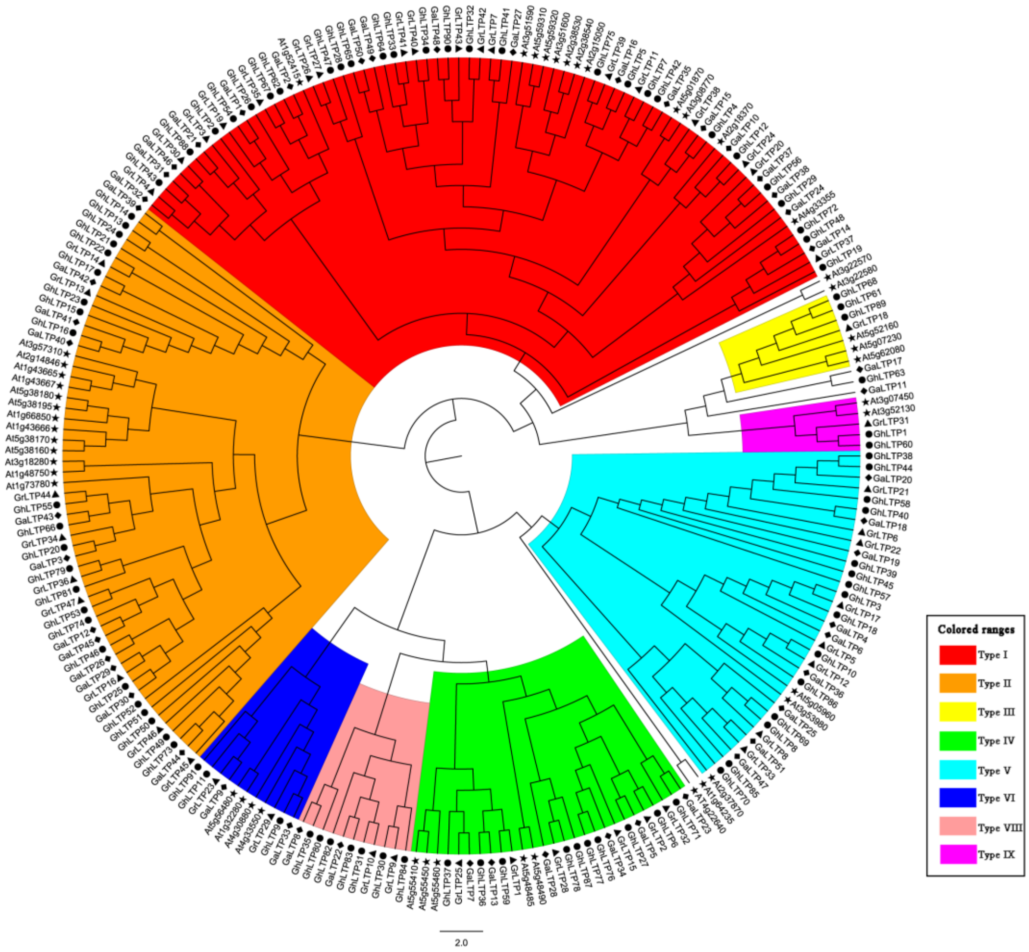

Figure S1: Phylogenetic relationships of the *nsLTPs* from *Gossypium* and Arabidopsis. Amino acid sequences were aligned using ClustalX and the maximum likelihood was generated with the PHYLIP software. The subfamilies within the *nsLTP* family are denoted by different colors.

a

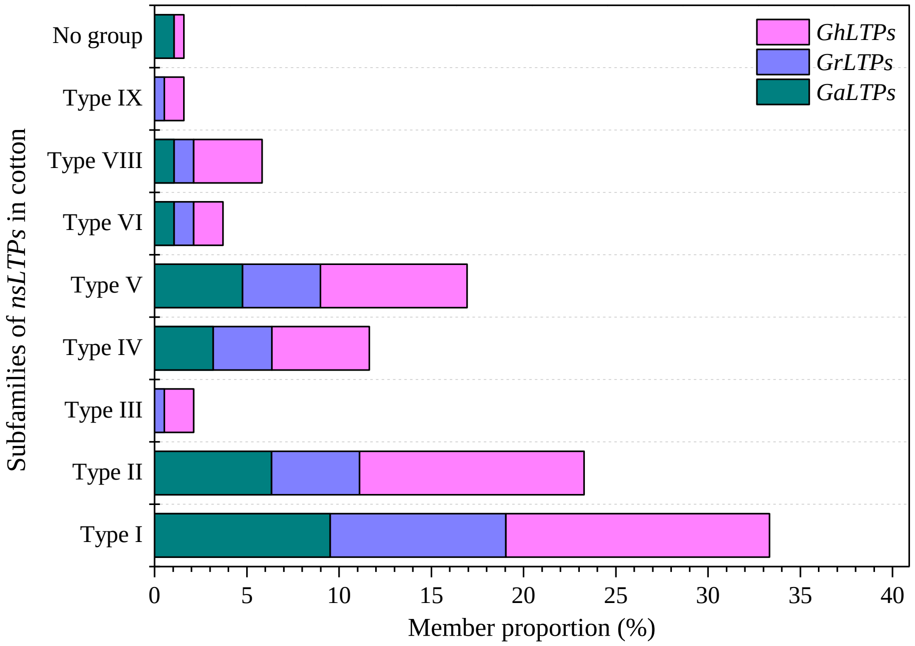

b

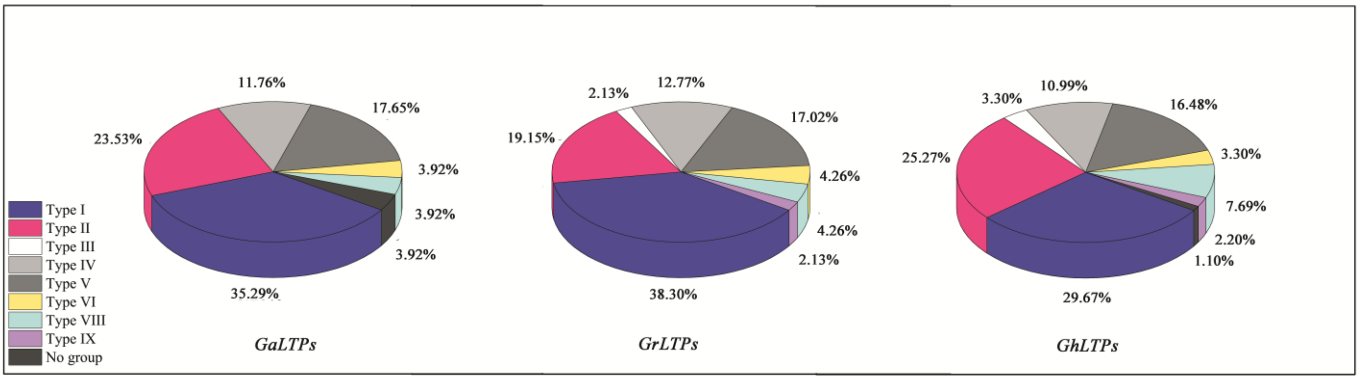

Figure S2: Distribution of *nsLTPs* within each subfamily in *G. arboretum*, *G. raimondii* and *G. hirsutum*. (a) The proportion of members in each *nsLTP* subfamily in *Gossypium*. (b) The percentage of members in each *nsLTP* subfamily in individual *Gossypium* species.

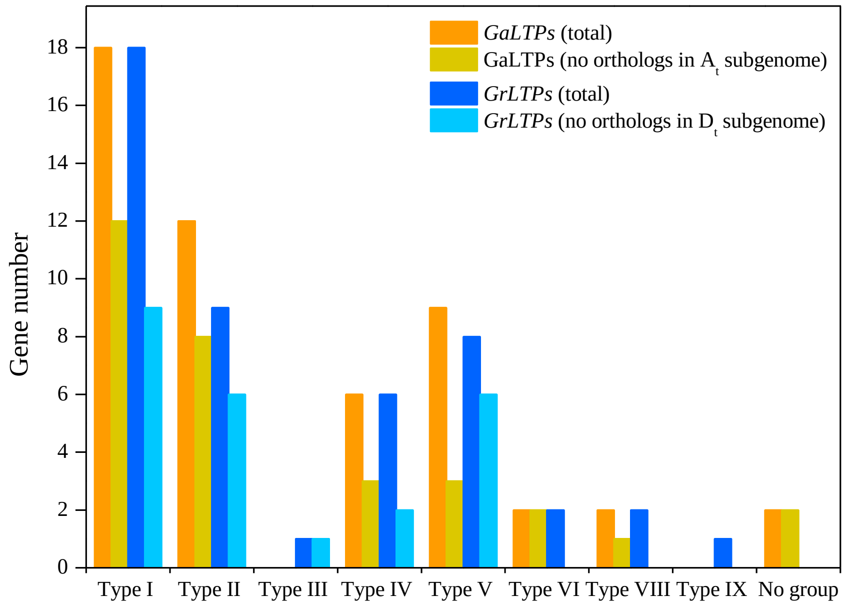

Figure S3: Distribution of the *GaLTPs* and *GrLTPs* with no orthologs existed in the A_t subgenome and D_t subgenome, respectively, of *G. hirsutum* within each subfamily.

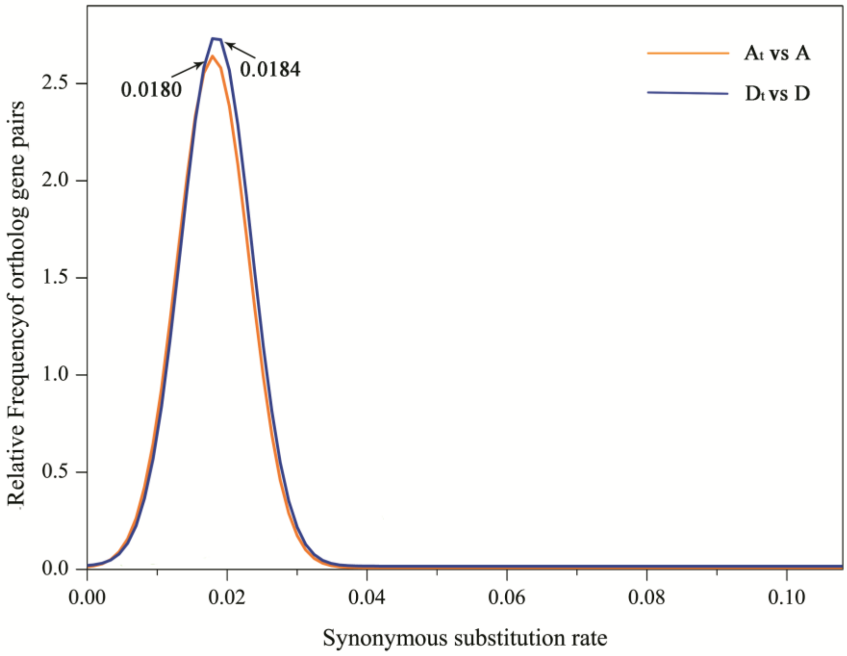

Figure S4: Distribution of Ks values for orthologous gene sets between the A_t subgenome in *G. hirsutum* and the A genome in *G. arboretum*, the D_t subgenome in *G. hirsutum* and the D genome in *G. raimondii*. Peak values for each comparison are indicated by arrows.

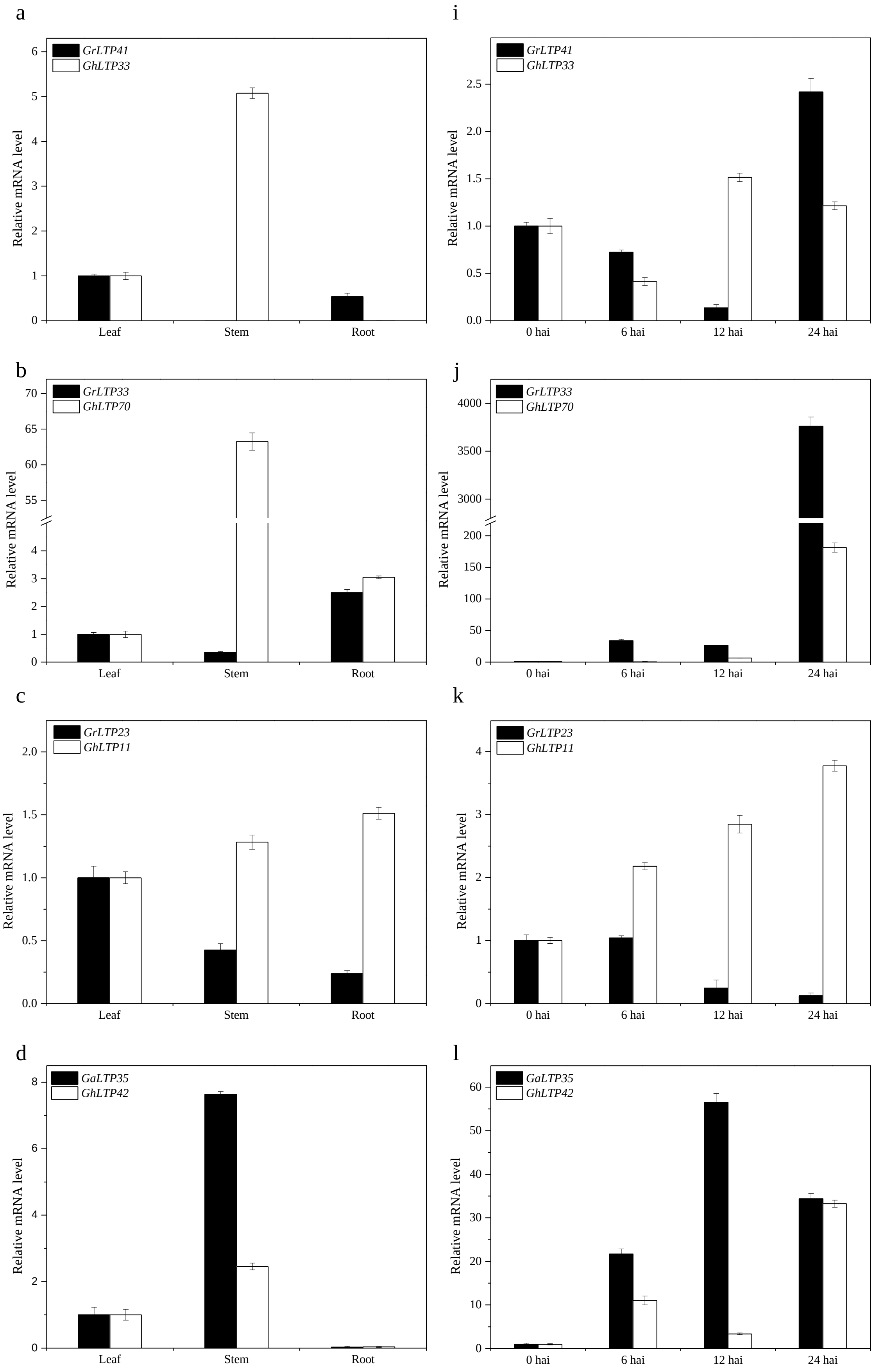
a
GrLTP41
GhLTP33
Relative mRNA level
0
1
2
3
4
5
6
Leaf
Stem
Root
i
GrLTP41
GhLTP33
Relative mRNA level
0.0
0.5
1.0
1.5
2.0
2.5
0 hai
6 hai
12 hai
24 hai
b
GrLTP33
GhLTP70
Relative mRNA level
0
1
2
3
4
55
60
65
70
Leaf
Stem
Root
j
GrLTP33
GhLTP70
Relative mRNA level
0
50
100
150
200
3000
3500
4000
0 hai
6 hai
12 hai
24 hai
c
GrLTP23
GhLTP11
Relative mRNA level
0.0
0.5
1.0
1.5
2.0
Leaf
Stem
Root
k
GrLTP23
GhLTP11
Relative mRNA level
0
1
2
3
4
0 hai
6 hai
12 hai
24 hai
d
GaLTP35
GhLTP42
Relative mRNA level
0
2
4
6
8
Leaf
Stem
Root
l
GaLTP35
GhLTP42
Relative mRNA level
0
10
20
30
40
50
60
0 hai
6 hai
12 hai
24 hai

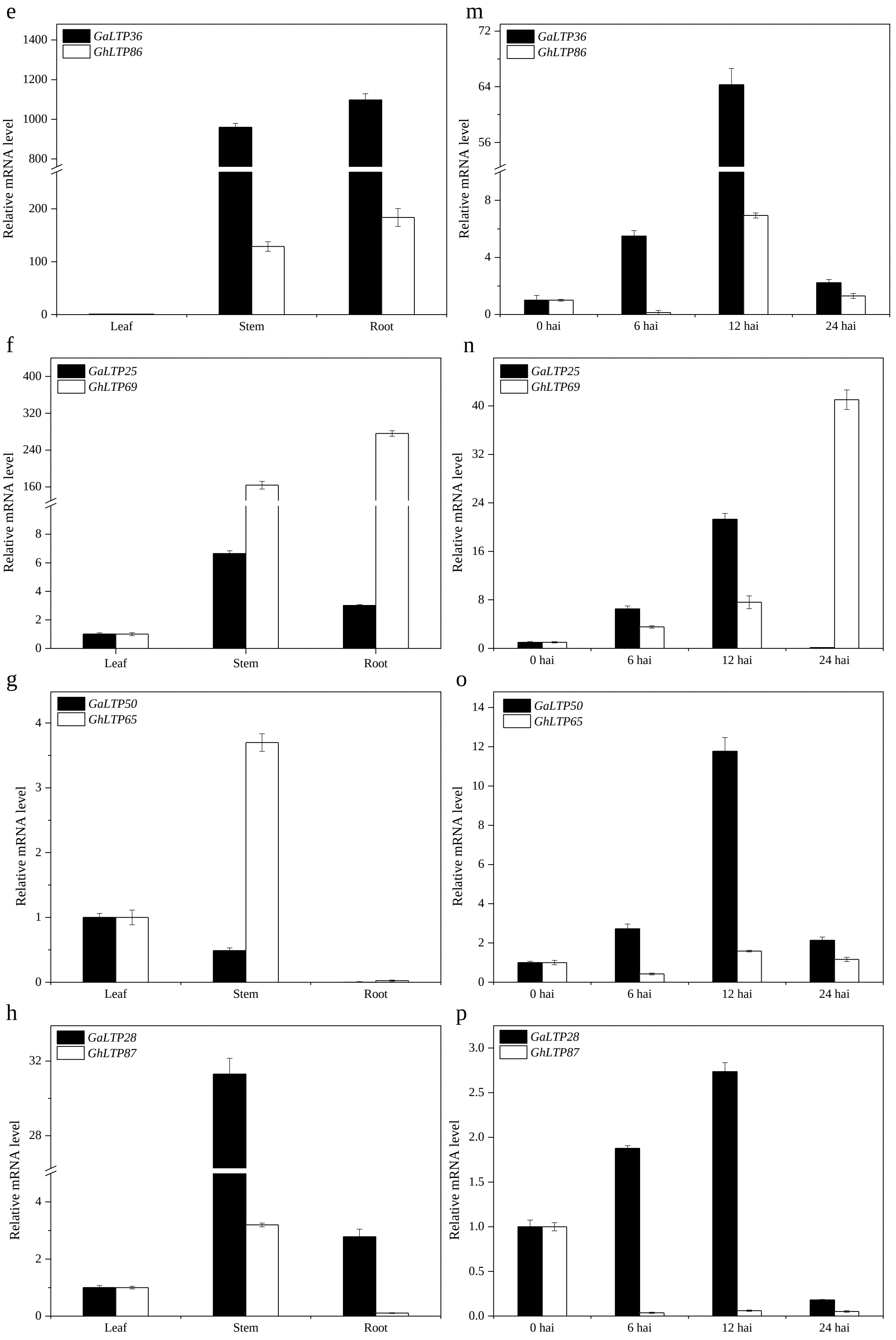

Figure S5: Expression analysis of interspecific orthologous gene pairs in different organs (a-h) and leaves after *V. dahliae* treatment (i-p).

Table S1: The structural analysis of nsLTPs identified in this study.

Name	Locus name	[a]SP ([b]AA)	[c]MP (AA)	MP ([d]Mw)	MP α-helix	MP Extended strand	MP β-turn	MP Random coil	MP Theoretical pI
GaLTP1	Cotton_A_00069	20	95	10787.33	55	6	5	29	5.16
GaLTP2	Cotton_A_01331	16	101	10701.13	29	28	7	37	3.89
GaLTP3	Cotton_A_01416	29	68	7307.64	12	13	4	39	9.44
GaLTP4	Cotton_A_02458	25	89	9094.51	35	11	6	37	8.48
GaLTP5	Cotton_A_03771	23	76	8049.32	15	8	4	49	4.03
GaLTP6	Cotton_A_05564	27	89	9237.67	25	17	10	37	8.2
GaLTP7	Cotton_A_06585	25	96	10721.49	24	11	2	59	8.39
GaLTP8	Cotton_A_07101	22	97	10270.80	32	10	6	49	7.68
GaLTP9	Cotton_A_07180	22	86	9331.06	31	19	10	26	9.22
GaLTP10	Cotton_A_07873	26	111	11538.38	46	17	8	40	8.96
GaLTP11	Cotton_A_08207	23	87	9073.77	29	19	10	25	9.22
GaLTP12	Cotton_A_11439	28	68	7116.32	12	16	3	37	9.15
GaLTP13	Cotton_A_12110	28	88	9767.75	15	23	2	48	9.73
GaLTP14	Cotton_A_12217	31	89	9276.66	42	9	7	31	4.81
GaLTP15	Cotton_A_12467	29	111	11498.75	55	7	8	41	4.32
GaLTP16	Cotton_A_12468	25	91	9692.22	38	13	11	29	10.59
GaLTP17	Cotton_A_13329	25	113	11722.52	36	19	6	52	4.5
GaLTP18	Cotton_A_13511	27	95	10468.19	27	16	12	40	8.97
GaLTP19	Cotton_A_13537	27	95	10416.08	31	15	10	39	8.83
GaLTP20	Cotton_A_13538	27	95	10468.19	28	16	11	40	8.97
GaLTP21	Cotton_A_15058	24	89	9196.73	39	14	5	31	8.49
GaLTP22	Cotton_A_17134	20	83	8851.46	39	12	6	26	8.83
GaLTP23	Cotton_A_17182	28	78	8483.73	22	10	13	33	7.68

GaLTP24	Cotton_A_17610	19	97	10308.82	41	20	4	32	5.78
GaLTP25	Cotton_A_20233	29	92	9213.1	26	11	11	44	0.02
GaLTP26	Cotton_A_20745	22	79	7116.32	12	16	3	37	9.15
GaLTP27	Cotton_A_21098	20	89	9063.49	29	4	12	44	9.08
GaLTP28	Cotton_A_21142	22	76	7949.48	11	8	9	48	8.72
GaLTP29	Cotton_A_21733	22	80	8340.79	10	6	4	60	8.18
GaLTP30	Cotton_A_21734	20	78	8309.79	22	8	7	41	7.64
GaLTP31	Cotton_A_22987	25	91	9989.64	32	12	14	33	5.44
GaLTP32	Cotton_A_22988	23	91	9536.35	22	15	10	44	9.01
GaLTP33	Cotton_A_23734	20	85	9045.65	22	16	10	37	5.18
GaLTP34	Cotton_A_24328	22	76	8180.7	14	15	9	38	7.47
GaLTP35	Cotton_A_24521	24	89	9341.87	42	8	3	36	9.26
GaLTP36	Cotton_A_25241	24	104	11270.12	28	23	9	44	9.15
GaLTP37	Cotton_A_26991	24	91	9735.2	49	8	6	28	6.44
GaLTP38	Cotton_A_26992	24	91	9680.19	38	10	5	38	5.62
GaLTP39	Cotton_A_27289	24	72	8107.59	38	2	3	29	8.81
GaLTP40	Cotton_A_27290	24	72	8106.54	38	6	3	25	8.44
GaLTP41	Cotton_A_27291	24	73	8163.59	39	6	2	26	8.44
GaLTP42	Cotton_A_27292	24	72	8018.39	38	2	3	29	8.44
GaLTP43	Cotton_A_27601	19	74	7782.11	17	18	6	33	9.33
GaLTP44	Cotton_A_30767	28	68	7364.64	13	11	4	40	9.02
GaLTP45	Cotton_A_30768	28	68	7116.32	12	16	3	37	9.15
GaLTP46	Cotton_A_34317	26	100	10423	39	12	7	42	4.61
GaLTP47	Cotton_A_35364	29	98	10143.19	36	11	13	38	10.34
GaLTP48	Cotton_A_38176	26	115	12523.61	42	31	5	37	9.4
GaLTP49	Cotton_A_39372	26	94	9304.75	23	16	10	45	9.2

GaLTP50	Cotton_A_39373	26	94	9315.73	26	14	6	48	9.07
GaLTP51	Cotton_A_39479	29	93	9531.45	34	6	15	38	10.34
GhLTP1	CotAD_01070	37	80	8531.08	27	7	5	41	8.5
GhLTP2	CotAD_01570	17	90	9270.76	45	16	6	23	8.2
GhLTP3	CotAD_03320	25	91	9368.87	35	13	7	36	8.69
GhLTP4	CotAD_10339	29	110	11397.69	53	7	9	41	4.39
GhLTP5	CotAD_10340	25	91	9722.24	35	13	11	32	10.59
GhLTP6	CotAD_10834	25	76	7976.27	16	10	4	46	4.03
GhLTP7	CotAD_11540	24	90	9439.05	40	11	5	34	9.28
GhLTP8	CotAD_13086	29	100	10207.13	33	9	11	47	9.78
GhLTP9	CotAD_16459	25	85	9045.65	22	16	10	37	5.18
GhLTP10	CotAD_17445	24	91	9671.16	21	19	8	43	8.7
GhLTP11	CotAD_17521	25	83	9073.77	29	19	10	25	9.22
GhLTP12	CotAD_17538	26	107	11025.83	41	23	6	37	9.26
GhLTP13	CotAD_18219	24	72	7116.32	12	16	3	37	9.15
GhLTP14	CotAD_18220	24	72	8045.50	37	2	3	30	8.81
GhLTP15	CotAD_18221	24	72	8004.36	39	2	3	28	8.44
GhLTP16	CotAD_18222	24	73	8191.65	39	7	2	25	8.44
GhLTP17	CotAD_18223	24	72	8018.39	38	2	3	29	8.44
GhLTP18	CotAD_18894	25	91	9425.93	38	11	4	38	8.69
GhLTP19	CotAD_20323	26	107	11358	30	17	7	53	7.67
GhLTP20	CotAD_21798	29	68	7307.64	12	13	4	39	9.44
GhLTP21	CotAD_23635	24	72	8034.39	37	2	3	30	8.44
GhLTP22	CotAD_23636	24	72	8018.39	38	2	3	29	8.44
GhLTP23	CotAD_23637	24	72	8034.39	37	2	3	30	8.44
GhLTP24	CotAD_23638	24	72	8149.5	42	2	2	26	8.49

GhLTP25	CotAD_24076	21	78	8311.77	19	9	6	44	7.64
GhLTP26	CotAD_24840	20	95	10704.2	51	8	5	31	5.44
GhLTP27	CotAD_25021	23	76	8049.32	15	8	4	49	4.03
GhLTP28	CotAD_25158	28	93	9920.55	30	13	3	47	8.41
GhLTP29	CotAD_26415	24	91	9648.12	43	11	5	32	5.62
GhLTP30	CotAD_27867	19	103	11241.12	36	20	7	40	4.27
GhLTP31	CotAD_27868	24	103	10779.59	27	14	13	49	8.51
GhLTP32	CotAD_27922	26	94	9264.68	27	15	7	45	9.16
GhLTP33	CotAD_27923	26	94	9330.75	22	17	9	46	9.18
GhLTP34	CotAD_27924	26	94	9065.34	20	17	9	48	8.87
GhLTP35	CotAD_28068	17	102	10964.48	31	16	4	51	6.05
GhLTP36	CotAD_29475	28	88	9733.73	15	21	4	48	9.73
GhLTP37	CotAD_29788	25	73	8032.75	20	3	2	48	8.79
GhLTP38	CotAD_29841	27	95	10482.22	26	16	13	40	8.97
GhLTP39	CotAD_29842	27	95	10463.18	27	18	10	40	8.99
GhLTP40	CotAD_29865	27	95	10468.19	27	16	12	40	8.97
GhLTP41	CotAD_30268	26	94	9584.02	33	8	15	38	8.9
GhLTP42	CotAD_30510	22	91	9544.08	42	10	3	36	9.26
GhLTP43	CotAD_32563	25	92	9949.8	26	13	14	39	8.19
GhLTP44	CotAD_34646	27	95	10468.19	27	16	12	40	8.97
GhLTP45	CotAD_34648	27	95	10466.24	37	11	10	37	9.24
GhLTP46	CotAD_34827	34	77	8309.49	32	6	5	34	4.77
GhLTP47	CotAD_35149	29	92	9806.39	29	16	2	45	8.4
GhLTP48	CotAD_35989	31	91	9501.99	42	12	7	30	5.17
GhLTP49	CotAD_38590	28	68	7352.67	19	9	4	36	9.16
GhLTP50	CotAD_38591	28	68	7361.68	19	9	4	36	9.16

GhLTP51	CotAD_38592	28	68	7411.74	18	10	4	36	9.3
GhLTP52	CotAD_38593	28	68	7383.69	16	6	5	41	9.3
GhLTP53	CotAD_38595	28	68	7060.26	8	15	2	43	9.02
GhLTP54	CotAD_39162	20	95	10759.28	55	6	4	30	5.16
GhLTP55	CotAD_39389	19	74	7854.17	17	18	6	33	9.18
GhLTP56	CotAD_39609	24	91	9751.24	51	10	4	26	6.44
GhLTP57	CotAD_40502	27	95	10507.27	35	11	13	36	9.1
GhLTP58	CotAD_40503	27	95	10507.27	35	11	13	36	9.1
GhLTP59	CotAD_41638	28	83	9222.07	13	21	0	49	9.78
GhLTP60	CotAD_42134	37	80	8503.02	27	8	5	40	8.5
GhLTP61	CotAD_45698	28	71	7552.77	34	9	0	28	7.71
GhLTP62	CotAD_45700	16	101	10701.13	29	28	7	37	3.89
GhLTP63	CotAD_47132	25	113	11722.52	36	19	6	52	4.5
GhLTP64	CotAD_47196	26	94	9221.61	30	17	11	36	9.03
GhLTP65	CotAD_47197	26	94	9273.65	28	14	10	42	9.07
GhLTP66	CotAD_52310	19	74	7782.11	17	18	6	33	9.33
GhLTP67	CotAD_52434	21	99	10569.12	27	30	8	34	4.28
GhLTP68	CotAD_52438	28	71	7509.74	34	10	0	27	7.71
GhLTP69	CotAD_52678	29	89	8914.76	26	8	11	44	10.15
GhLTP70	CotAD_54258	29	103	10532.51	36	10	15	42	10.17
GhLTP71	CotAD_55610	28	78	8481.80	26	9	12	31	8.18
GhLTP72	CotAD_55901	31	91	9517.99	42	11	7	31	5.17
GhLTP73	CotAD_57885	28	68	7364.64	13	11	4	40	9.02
GhLTP74	CotAD_57886	28	68	7102.25	12	16	6	34	9.02
GhLTP75	CotAD_60214	25	91	9677.2	45	12	7	27	10.34
GhLTP76	CotAD_60923	22	87	9562.26	18	22	6	41	5.48

GhLTP77	CotAD_61486	22	76	8180.70	14	15	9	38	7.47
GhLTP78	CotAD_62024	25	76	7949.48	11	8	9	48	8.72
GhLTP79	CotAD_62387	28	68	7548.81	20	12	2	34	8.7
GhLTP80	CotAD_63271	22	90	9772.28	41	10	6	33	5.15
GhLTP81	CotAD_63864	28	68	7505.79	21	11	2	34	8.48
GhLTP82	CotAD_64828	23	84	9037.52	35	9	8	32	6.17
GhLTP83	CotAD_64829	24	83	8885.48	31	12	8	32	8.83
GhLTP84	CotAD_64830	19	98	10221.75	31	21	6	40	3.53
GhLTP85	CotAD_66200	29	92	9368.33	36	8	13	35	10.72
GhLTP86	CotAD_68865	24	91	9643.11	26	16	7	42	8.7
GhLTP87	CotAD_71505	22	76	7922.45	11	9	9	47	8.72
GhLTP88	CotAD_74405	24	90	9378.95	44	14	7	25	8.71
GhLTP89	CotAD_74574	33	67	7064.03	21	4	1	41	5.38
GhLTP90	CotAD_75312	26	94	10047.86	38	24	3	29	9.59
GhLTP91	CotAD_76539	26	86	8917.52	22	16	11	35	5.18
GrLTP1	Cotton_D_gene_10001136	28	83	9222.07	13	21	0	49	9.78
GrLTP2	Cotton_D_gene_10002491	25	76	7976.27	16	10	4	46	4.03
GrLTP3	Cotton_D_gene_10003092	17	90	9379.94	45	13	6	26	8.49
GrLTP4	Cotton_D_gene_10004032	23	91	9734.57	24	15	11	41	9.02
GrLTP5	Cotton_D_gene_10004247	27	91	9581.1	28	16	5	42	8.49
GrLTP6	Cotton_D_gene_10004792	27	112	12638.79	34	26	14	38	9.07
GrLTP7	Cotton_D_gene_10006582	26	94	9407.85	32	6	12	43	8.91
GrLTP8	Cotton_D_gene_10007616	29	89	8880.74	27	8	11	43	10.15
GrLTP9	Cotton_D_gene_10009769	20	98	10769.56	35	16	7	40	4.27
GrLTP10	Cotton_D_gene_10009771	24	116	12102.07	44	10	13	49	5.63
GrLTP11	Cotton_D_gene_10011175	24	90	9439.05	40	11	5	34	9.28

GrLTP12	Cotton_D_gene_10012090	24	91	9643.11	25	16	7	43	8.7
GrLTP13	Cotton_D_gene_10012564	24	72	8048.41	38	2	3	29	8.44
GrLTP14	Cotton_D_gene_10012565	24	72	8048.41	38	2	3	29	8.42
GrLTP15	Cotton_D_gene_10014066	22	76	8211.67	17	13	5	41	5.13
GrLTP16	Cotton_D_gene_10016400	25	72	7580.03	13	3	3	53	8.69
GrLTP17	Cotton_D_gene_10017219	25	91	9398.9	32	13	7	39	8.69
GrLTP18	Cotton_D_gene_10017840	35	64	6652.7	21	6	0	37	5.99
GrLTP19	Cotton_D_gene_10018004	20	95	10759.28	55	6	4	30	5.16
GrLTP20	Cotton_D_gene_10020138	24	91	9988.45	44	11	7	33	5.16
GrLTP21	Cotton_D_gene_10021595	27	95	10468.19	27	16	12	40	8.97
GrLTP22	Cotton_D_gene_10021630	27	95	10523.23	332	12	11	40	8.97
GrLTP23	Cotton_D_gene_10024327	22	91	9960.86	33	19	7	32	9.33
GrLTP24	Cotton_D_gene_10024343	25	92	9196.65	38	13	6	35	9.02
GrLTP25	Cotton_D_gene_10024928	25	81	8879.54	22	4	4	51	8.78
GrLTP26	Cotton_D_gene_10025353	29	98	10470.2	35	16	2	45	8.4
GrLTP27	Cotton_D_gene_10025357	29	98	10470.2	35	16	2	45	8.4
GrLTP28	Cotton_D_gene_10026448	22	76	7949.48	11	8	9	48	8.72
GrLTP29	Cotton_D_gene_10028428	20	84	9108.66	22	19	10	33	5.18
GrLTP30	Cotton_D_gene_10028488	26	100	10438.95	37	11	9	43	4.47
GrLTP31	Cotton_D_gene_10029118	37	80	8531.08	27	7	5	41	8.5
GrLTP32	Cotton_D_gene_10029520	28	78	8438.77	25	10	12	31	8.18
GrLTP33	Cotton_D_gene_10029873	29	103	10581.59	36	14	15	38	10.09
GrLTP34	Cotton_D_gene_10030639	29	68	7992.43	13	18	5	39	9.41
GrLTP35	Cotton_D_gene_10030724	22	101	10672.2	26	30	8	37	4.07
GrLTP36	Cotton_D_gene_10034860	28	68	7479.71	17	12	2	37	8.48
GrLTP37	Cotton_D_gene_10035351	31	89	9246.63	42	10	7	30	4.81

GrLTP38	Cotton_D_gene_10035553	29	111	11634.97	50	8	8	45	4.43
GrLTP39	Cotton_D_gene_10035554	25	91	9677.2	44	12	8	27	10.34
GrLTP40	Cotton_D_gene_10036318	26	94	9091.42	20	18	10	46	8.87
GrLTP41	Cotton_D_gene_10036321	26	94	9330.75	22	17	9	46	9.18
GrLTP42	Cotton_D_gene_10036322	26	98	9767.21	28	18	7	45	9.18
GrLTP43	Cotton_D_gene_10036323	26	98	10419.16	35	20	3	40	9.44
GrLTP44	Cotton_D_gene_10040151	19	74	7854.17	17	18	6	33	9.18
GrLTP45	Cotton_D_gene_10040242	28	68	7362.71	13	8	4	43	9.16
GrLTP46	Cotton_D_gene_10040245	28	68	7352.67	19	8	4	37	9.16
GrLTP47	Cotton_D_gene_10040247	28	68	7060.26	13	9	4	42	9.02

[a]SP, signal peptide.

[b]AA, number of amino acids.

[c]MP, mature protein.

[d]Mw, molecular weight in Dalton.

Table S2: Putative *nsLTPs* identified in *G. raimondii*, *G. arboretum* and *G. hirsutum*.

Name	Subfamily	Arabidopsis ortholog	E-value	Extron number	Intron number	Chomosome	Start (bp)	End (bp)
GaLTP1	Type I	*At2g150502*	1.00E-10	1	0	Chr7	37790084	37790431
GaLTP2	Type I	*At3g51590*	1.00E-11	1	0	Chr6	120892850	120893203
GaLTP3	Type II	*At3g53980*	3.00E-53	1	0	Chr6	120200194	120200484
GaLTP4	Type V	*At3g53980*	3.00E-53	1	0	Chr11	21232372	21232716
GaLTP5	Type IV	*At5g48490*	3.00E-24	1	0	Chr4	51857071	51857370
GaLTP6	Type V	*At3g53980*	9.00E-50	1	0	Chr4	129150615	129150965
GaLTP7	Type IV	*At5g55410*	1.00E-21	2	1	Chr7	27747819	27748309
GaLTP8	Type VIII	*At5g48485*	0.002	2	1	Chr6	74748415	74748911
GaLTP9	Type VI	*At5g484852*	3.00E-09	2	1	Chr6	73812163	73812588
GaLTP10	Type I	*At2g18370*	7.00E-25	1	0	Chr6	50966513	50966926
GaLTP11	No group	*At3g22580*	6.00E-08	1	0	Chr4	26385953	26386285
GaLTP12	Type II	*At3g18280*	8.00E-31	1	0	Chr6	78902349	78902639
GaLTP13	Type IV	*At5g55410*	9.00E-22	1	0	Chr10	26725539	26725889
GaLTP14	Type I	*At2g150501*	9.00E-16	1	0	Chr1	84235317	84235679
GaLTP15	Type I	*At3g08770*	2.00E-30	2	1	Chr11	8413686	8414211
GaLTP16	Type I	*At2g38540*	2.00E-34	2	1	Chr11	8407512	8407959
GaLTP17	No group	*At2g15050*	0.019	2	1	Chr1	70399019	70399471
GaLTP18	Type V	*At5g05960*	3.00E-26	2	1	Chr10	96995648	96996131
GaLTP19	Type V	*At5g05960*	4.00E-25	2	1	Chr10	96682768	96683257
GaLTP20	Type V	*At5g05960*	3.00E-26	2	1	Chr10	96658053	96658518
GaLTP21	Type I	*At3g51590*	1.00E-12	2	1	Chr10	87747661	87748001
GaLTP22	Type VIII	*At3g22580*	0.003	1	0	Chr7	110981628	110981939
GaLTP23	Type IV	*At5g48490*	2.00E-15	1	0	Chr13	70584544	70584864
GaLTP24	Type I	*At3g51600*	2.00E-18	1	0	Chr12	45812028	45812378

GaLTP25	Type V	*At2g37870*	1.00E-45	2	1	Chr6	23061702	23063479
GaLTP26	Type II	*At3g18280*	1.00E-24	1	0	Chr10	10355691	10355993
GaLTP27	Type I	*At2g150501*	2.00E-25	2	1	Chr11	92194248	92194575
GaLTP28	Type IV	*At5g48485*	2.00E-15	1	0	Chr9	41574634	41574930
GaLTP29	Type II	*At3g18280*	2.00E-19	1	0	Chr8	39561277	39561585
GaLTP30	Type II	*At1g48750*	1.00E-22	1	0	Chr8	39566484	39566780
GaLTP31	Type I	*At2g150502*	8.00E-09	2	1	Chr3	4279566	4280421
GaLTP32	Type I	*At4g33355*	1.00E-11	1	0	Chr3	4276288	4276632
GaLTP33	Type VI	*At5g484851*	5.00E-22	2	1	Chr1	108554141	108554563
GaLTP34	Type IV	*At5g48490*	3.00E-22	1	0	Chr1	103126087	103126383
GaLTP35	Type I	*At2g38540*	2.00E-33	1	0	Chr5	56831952	56832293
GaLTP36	Type V	*At5g05960*	3.00E-47	1	0	Chr12	18135205	18135591
GaLTP37	Type I	*At4g33355*	2.00E-16	1	0	Chr13	24147133	24147480
GaLTP38	Type I	*At4g33355*	6.00E-17	1	0	Chr13	24140600	24140947
GaLTP39	Type II	*At3g18280*	5.00E-09	1	0	Chr6	94955734	94956024
GaLTP40	Type II	*At3g18280*	2.00E-07	1	0	Chr6	94959267	94959557
GaLTP41	Type II	*At3g18280*	5.00E-09	1	0	Chr6	94965685	94965978
GaLTP42	Type II	*At3g18280*	2.00E-07	1	0	Chr6	94969172	94969462
GaLTP43	Type II	*At3g18280*	2.00E-32	1	0	Chr11	67867436	67867717
GaLTP44	Type II	*At3g18280*	3.00E-31	1	0	Chr1	8252668	8252958
GaLTP45	Type II	*At3g18280*	8.00E-31	1	0	Chr1	8301176	8301466
GaLTP46	Type I	*At3g08770*	2.00E-08	2	1	Chr1	35339658	35340131
GaLTP47	Type V	*At2g37870*	1.00E-44	1	0	Chr7	6217746	6218129
GaLTP48	Type I	*At2g38540*	5.00E-24	2	1	Chr9	8230584	8231141
GaLTP49	Type I	*At2g38540*	5.00E-36	2	1	Chr1	61844160	61844602
GaLTP50	Type I	*At2g150502*	9.00E-37	2	1	Chr1	61859499	61859941

GaLTP51	Type V	*At2g37870*	6.00E-44	1	0	Chr4	19907570	19907935
GhLTP1	Type IX	*At3g52130*	2.00E-36	2	1	Dt_chr10	7995890	7996345
GhLTP2	Type I	*At3g51590*	3.00E-15	1	0	Dt_chr9	60156147	60156470
GhLTP3	Type V	*At3g53980*	3.00E-57	2	1	Dt_chr6	7697879	7698311
GhLTP4	Type I	*At3g08770*	9.00E-30	2	1	scaffold251.1	1210728	1211250
GhLTP5	Type I	*At2g38540*	1.00E-33	2	1	scaffold251.1	1216963	1217410
GhLTP6	Type IV	*At5g48490*	8.00E-23	1	0	Dt_chr7	11987749	11988054
GhLTP7	Type I	*At2g38540*	1.00E-33	2	1	scaffold189.1	539409	539875
GhLTP8	Type V	*At2g37870*	6.00E-43	2	1	Dt_chr8	43220389	43220827
GhLTP9	Type VI	*At5g484851*	1.00E-21	2	1	Dt_chr1	52500650	52501305
GhLTP10	Type V	*At5g05960*	2.00E-49	2	1	At_chr7	12044173	12044870
GhLTP11	Type VI	*At5g484852*	1.00E-08	2	1	Dt_chr8	49107411	49107836
GhLTP12	Type I	*At2g18370*	2.00E-29	2	1	Dt_chr8	49291720	49292202
GhLTP13	Type II	*At3g18280*	8.00E-07	1	0	scaffold377.1	484732	485022
GhLTP14	Type II	*At3g18280*	7.00E-09	1	0	scaffold377.1	509762	510052
GhLTP15	Type II	*At3g18280*	6.00E-07	1	0	scaffold377.1	513317	513607
GhLTP16	Type II	*At3g18280*	2.00E-09	1	0	scaffold377.1	519521	519814
GhLTP17	Type II	*At3g18280*	3.00E-07	1	0	scaffold377.1	523004	523294
GhLTP18	Type V	*At3g53980*	2.00E-55	2	1	At_chr6	55578407	55578839
GhLTP19	Type I	*At2g18370*	2.00E-12	2	1	Dt_chr6	19314266	19315520
GhLTP20	Type II	*At3g18280*	1.00E-29	1	0	scaffold506.1	479578	479871
GhLTP21	Type II	*At3g18280*	9.00E-07	1	0	Dt_chr6	5322358	5322648
GhLTP22	Type II	*At3g18280*	6.00E-07	1	0	Dt_chr6	5318885	5319175
GhLTP23	Type II	*At3g18280*	6.00E-06	1	0	Dt_chr6	5309137	5309427
GhLTP24	Type II	*At3g18280*	2.00E-07	1	0	Dt_chr6	5283498	5283788
GhLTP25	Type II	*At1g48750*	2.00E-22	1	0	Dt_chr5	9566262	9566561

GhLTP26	Type I	*At2g150503*	6.00E-13	1	0	scaffold783.1	589921	590268
GhLTP27	Type IV	*At5g48490*	5.00E-24	1	0	At_chr7	2804588	2805287
GhLTP28	Type I	*At4g33355*	5.00E-22	2	1	At_chr9	36696240	36696704
GhLTP29	Type I	*At4g33355*	4.00E-16	1	0	Dt_chr13	46184850	46185197
GhLTP30	Type VIII	*At3g22580*	0.002	1	0	Dt_chr13	12592021	12592389
GhLTP31	Type VIII	*At3g22580*	0.16	3	2	Dt_chr13	12597257	12599015
GhLTP32	Type I	*At2g38540*	4.00E-35	2	1	Dt_chr11	17862337	17862825
GhLTP33	Type I	*At2g15050*	7.00E-22	2	1	Dt_chr11	17854681	17855123
GhLTP34	Type I	*At2g38540*	1.00E-28	2	1	Dt_chr11	17821616	17822058
GhLTP35	Type VIII	*At3g18280*	0.002	2	1	Dt_chr8	51926832	51927319
GhLTP36	Type IV	*At5g55410*	9.00E-20	1	0	scaffold743.1	599210	599560
GhLTP37	Type IV	*At5g55410*	2.00E-15	2	1	Dt_chr2	45146789	45147191
GhLTP38	Type V	*At5g05960*	5.00E-26	2	1	At_chr4	88619547	88620012
GhLTP39	Type V	*At5g05960*	6.00E-25	2	1	At_chr4	88637685	88638174
GhLTP40	Type V	*At5g05960*	5.00E-26	2	1	At_chr4	88880933	88881416
GhLTP41	Type I	*At2g38540*	2.00E-27	2	1	Dt_chr6	15952855	15953336
GhLTP42	Type I	*At2g38540*	1.00E-32	1	0	At_chr9	4029328	4029669
GhLTP43	Type I	*At2g150502*	3.00E-10	1	0	scaffold417.1	810840	811193
GhLTP44	Type V	*At5g05960*	6.00E-26	2	1	scaffold1227.1	461720	462203
GhLTP45	Type V	*At3g53980*	6.00E-29	2	1	scaffold1227.1	487660	488136
GhLTP46	Type II	*At1g48750*	2.00E-23	1	0	At_chr9	34037734	34038069
GhLTP47	Type I	*At4g33355*	3.00E-23	2	1	scaffold1451.1	395908	396371
GhLTP48	Type I	*At2g18370*	3.00E-16	2	1	Dt_chr1	44631070	44631801
GhLTP49	Type II	*At1g48750*	1.00E-22	1	0	At_chr2	58039220	58039510
GhLTP50	Type II	*At1g48750*	5.00E-21	1	0	At_chr2	58064775	58065065
GhLTP51	Type II	*At1g48750*	5.00E-21	1	0	At_chr2	58084053	58084343

GhLTP52	Type II	*At1g48750*	1.00E-19	1	0	At_chr2	58113374	58113664
GhLTP53	Type II	*At1g48750*	4.00E-22	1	0	At_chr2	58137045	58137335
GhLTP54	Type I	*At2g150503*	3.00E-13	1	0	At_chr9	83561136	83561483
GhLTP55	Type II	*At3g18280*	4.00E-33	1	0	Dt_chr6	25357440	25357721
GhLTP56	Type I	*At2g150502*	7.00E-15	1	0	At_chr4	3446379	3446726
GhLTP57	Type V	*At5g05960*	2.00E-26	2	1	scaffold1251.1	148083	148548
GhLTP58	Type V	*At5g05960*	6.00E-27	2	1	scaffold1251.1	157882	158347
GhLTP59	Type IV	*At5g55410*	9.00E-20	1	0	Dt_chr9	1904516	1904851
GhLTP60	Type IX	*At3g52130*	6.00E-37	2	1	scaffold349.1	347450	347904
GhLTP61	Type III	*At5g52160*	3.00E-20	1	0	scaffold1695.1	22045	22344
GhLTP62	Type I	*At3g51590*	2.00E-11	2	1	scaffold1695.1	37989	38406
GhLTP63	No group	*At2g15050*	0.035	2	1	Dt_chr1	31430870	31431322
GhLTP64	Type I	*At2g38540*	4.00E-36	2	1	At_chr11	23618623	23619065
GhLTP65	Type I	*At2g150502*	9.00E-37	2	1	At_chr11	23633967	23634409
GhLTP66	Type II	*At3g18280*	3.00E-32	1	0	At_chr6	21954384	21954665
GhLTP67	Type I	*At2g150500*	4.00E-11	2	1	Dt_chr8	55438018	55438433
GhLTP68	Type III	*At5g52160*	2.00E-20	1	0	Dt_chr8	55427029	55427328
GhLTP69	Type V	*At2g37870*	2.00E-42	1	0	At_chr2	36952988	36953344
GhLTP70	Type V	*At2g37870*	8.00E-43	1	0	Dt_chr2	22181830	22182228
GhLTP71	Type IV	*At5g48490*	1.00E-12	1	0	At_chr13	85884728	85885048
GhLTP72	Type I	*At2g150501*	6.00E-17	2	1	scaffold1609.1	15679	16157
GhLTP73	Type II	*At3g18280*	5.00E-31	1	0	At_chr8	35649286	35649576
GhLTP74	Type II	*At3g18280*	5.00E-30	1	0	At_chr8	35696326	35696616
GhLTP75	Type I	*At2g38540*	8.00E-35	2	1	scaffold3642.1	106001	106453
GhLTP76	Type IV	*At5g48490*	8.00E-26	1	0	Dt_chr1	63922025	63922804
GhLTP77	Type IV	*At5g48490*	7.00E-22	1	0	Dt_chr1	4470224	4471096

GhLTP78	Type IV	*At5g48485*	5.00E-15	1	0	Dt_chr11	21104387	21104692
GhLTP79	Type II	*At3g18280*	5.00E-25	1	0	Dt_chr1	73461042	73461332
GhLTP80	Type VIII	*At5g48485*	0.005	1	0	scaffold3185.1	23443	23781
GhLTP81	Type II	*At3g18280*	5.00E-25	1	0	At_chr7	20929138	20929428
GhLTP82	Type VIII	*AtLtp2.14*	0.014	1	0	At_chr5	99739	100062
GhLTP83	Type VIII	*At3g22580*	0.007	1	0	At_chr5	106628	106951
GhLTP84	Type VIII	*At3g22580*	0.004	3	2	At_chr5	114483	116350
GhLTP85	Type V	*At2g37870*	1.00E-45	2	1	scaffold1812.1	298432	300037
GhLTP86	Type V	*At5g05960*	2.00E-50	2	1	At_chr9	8509131	8509592
GhLTP87	Type IV	*At5g48485*	5.00E-15	1	0	At_chr11	29312807	29313103
GhLTP88	Type I	*At3g51590*	4.00E-15	1	0	scaffold5357.1	12114	12458
GhLTP89	Type III	*At5g52160*	1.00E-25	1	0	Dt_chr6	33131085	33131387
GhLTP90	Type I	*At2g150502*	2.00E-24	2	1	At_chr11	19372247	19372745
GhLTP91	Type VI	*At5g484852*	2.00E-07	1	0	scaffold4801.1	43417	43755
GrLTP1	Type IV	*At5g55410*	5.00E-20	1	0	scaffold435	123202	123537
GrLTP2	Type IV	*At5g48490*	1.00E-22	1	0	Chr7	40432720	40433398
GrLTP3	Type I	*At3g51590*	2.00E-15	1	0	Chr9	9607173	9607496
GrLTP4	Type I	*At4g33355*	4.00E-12	1	0	scaffold371	334705	335049
GrLTP5	Type V	*At3g53980*	3.00E-54	2	1	scaffold286	482320	482767
GrLTP6	Type V	*At5g05960*	6.00E-26	1	0	Chr2	8222530	8222949
GrLTP7	Type I	*At2g38540*	3.00E-27	2	1	Chr6	24433348	24433829
GrLTP8	Type V	*At2g37870*	4.00E-43	1	0	scaffold182	509681	510037
GrLTP9	Type VIII	*At3g22580*	0.006	1	0	Chr3	1836681	1837037
GrLTP10	Type VIII	*At3g22570*	0.73	3	2	Chr3	1849058	1850292
GrLTP11	Type I	*At2g38540*	7.00E-34	2	1	Chr5	1501335	1502257
GrLTP12	Type V	*At5g05960*	4.00E-50	2	1	Chr12	19313246	19313956

GrLTP13	Type II	*At3g18280*	3.00E-07	1	0	Chr6	42840183	42840473
GrLTP14	Type II	*At3g18280*	4.00E-07	1	0	Chr6	42852799	42853089
GrLTP15	Type IV	*At5g48490*	1.00E-27	1	0	Chr5	6623886	6624972
GrLTP16	Type II	*At3g18280*	1.00E-18	1	0	Chr10	4014748	4015399
GrLTP17	Type V	*At3g53980*	3.00E-57	2	1	Chr6	7486974	7487406
GrLTP18	Type III	*At5g52160*	8.00E-24	1	0	Chr7	54617942	54618241
GrLTP19	Type I	*At2g150503*	6.00E-14	1	0	Chr8	21159872	21160219
GrLTP20	Type I	*At4g33355*	2.00E-16	1	0	Chr7	37404699	37405046
GrLTP21	Type V	*At5g05960*	3.00E-26	2	1	Chr11	44902634	44903121
GrLTP22	Type V	*At5g05960*	2.00E-26	2	1	Chr11	45341098	45341600
GrLTP23	Type VI	*At5g484852*	3.00E-09	1	0	Chr8	12195962	12196303
GrLTP24	Type I	*At2g18370*	3.00E-31	2	1	Chr8	12365204	12365642
GrLTP25	Type IV	*At5g55410*	7.00E-21	2	1	Chr2	39519217	39520164
GrLTP26	Type I	*At2g150502*	4.00E-22	1	0	Chr13	2918739	2919122
GrLTP27	Type I	*At2g150502*	4.00E-22	1	0	Chr13	2930157	2930540
GrLTP28	Type IV	*At5g48485*	2.00E-15	1	0	Chr11	21929397	21929693
GrLTP29	Type VI	*At5g484851*	2.00E-23	1	0	Chr1	19117567	19117881
GrLTP30	Type I	*At3g08770*	2.00E-09	2	1	Chr1	15688445	15688917
GrLTP31	Type IX	*At3g52130*	2.00E-36	2	1	Chr4	7802709	7803165
GrLTP32	Type IV	*At5g48490*	1.00E-11	1	0	Chr13	38141377	38141697
GrLTP33	Type V	*At2g37870*	8.00E-43	1	0	Chr8	16148423	16148821
GrLTP34	Type II	*At3g18280*	6.00E-30	1	0	Chr8	2437561	2438231
GrLTP35	Type I	*At3g51590*	3.00E-11	1	0	Chr8	3083653	3084024
GrLTP36	Type II	*At3g18280*	1.00E-25	1	0	Chr7	20469757	20470047
GrLTP37	Type I	*At4g33355*	8.00E-16	1	0	Chr9	39750022	39750384
GrLTP38	Type I	*At3g08770*	2.00E-31	2	1	Chr7	42467263	42467788

GrLTP39	Type I	*At2g38540*	4.00E-35	2	1	Chr7	42473453	42473885
GrLTP40	Type I	*At2g38540*	5.00E-29	2	1	Chr11	18001387	18001829
GrLTP41	Type I	*At2g150502*	1.00E-33	2	1	Chr11	18033941	18034759
GrLTP42	Type I	*At2g38540*	3.00E-34	1	0	Chr11	18041958	18042332
GrLTP43	Type I	*At2g38540*	2.00E-24	1	0	Chr11	18083522	18083896
GrLTP44	Type II	*At3g18280*	7.00E-33	1	0	Chr6	16341482	16342125
GrLTP45	Type II	*At1g48750*	6.00E-22	1	0	Chr8	41013956	41014246
GrLTP46	Type II	*At1g48750*	2.00E-22	1	0	Chr8	41041013	41041303
GrLTP47	Type II	*At1g48750*	2.00E-22	1	0	Chr8	41076321	41076611

Table S3: List of the orthologous groups of nsLTPs through OtrhoMCL clustering.

Name	Subfamily	Orthomcl_group	Seq_id_of_best_hit	E_value_mantissa	E_value_exponent	Identity(%)	Match(%)
GaLTP1	Type I	OG5_178339	rcom|29983.m003153	1	-22	43	87
GaLTP2	Type I	OG5_211688	rcom|30147.m014290	1	-31	59	91
GaLTP3	Type II	OG5_170522	rcom|30066.m000748	3	-28	64	91
GaLTP4	Type V	OG5_140479	rcom|30143.m001220	9	-39	66	100
GaLTP5	Type IV	OG5_150363	rcom|30178.m000847	2	-30	72	83
GaLTP6	Type V	OG5_140479	rcom|30143.m001220	7	-38	63	97
GaLTP7	Type IV	OG5_164277	atha|NP_200352	2	-18	37	100
GaLTP8	Type VIII	OG5_177605	rcom|30190.m011003	8	-20	46	84
GaLTP9	Type VI	OG5_244951	rcom|29738.m001007	8	-38	65	98
GaLTP10	Type I	OG5_212196	rcom|29616.m000215	1	-32	66	98
GaLTP11	No group	OG5_150394	rcom|29703.m001499	4	-34	67	92
GaLTP12	Type II	OG5_170522	atha|NP_188456	6	-25	54	100
GaLTP13	Type IV	OG5_164277	atha|NP_001032078	4	-18	40	100
GaLTP14	Type I	OG5_179243	rcom|30005.m001268	6	-27	45	98
GaLTP15	Type I	OG5_212357	atha|NP_187489	7	-23	49	84
GaLTP16	Type I	OG5_135466	rcom|29915.m000465	2	-29	55	96
GaLTP17	No group	OG5_170058	rcom|29942.m000735	3	-26	57	75
GaLTP18	Type V	OG5_140479	rcom|30143.m001220	4	-27	48	97
GaLTP19	Type V	OG5_140479	rcom|30143.m001220	2	-25	47	97
GaLTP20	Type V	OG5_140479	rcom|30143.m001220	5	-26	48	97
GaLTP21	Type I	OG5_244785	rcom|27996.m000149	1	-35	74	85
GaLTP22	Type VIII	OG5_177605	rcom|30190.m011003	2	-11	39	93
GaLTP23	Type IV	OG5_150363	rcom|29851.m002445	7	-16	52	82
GaLTP24	Type I	OG5_156310	rcom|30174.m008763	8	-29	53	97

GaLTP25	Type V	OG5_213111	rcom|29780.m001377	5	-43	71	100
GaLTP26	Type II	OG5_170522	atha|NP_188456	4	-20	48	91
GaLTP27	Type I	OG5_135466	rcom|29915.m000465	7	-28	54	99
GaLTP28	Type IV	OG5_150363	rcom|29888.m000322	2	-32	70	94
GaLTP29	Type II	OG5_170522	atha|NP_188456	2	-16	51	80
GaLTP30	Type II	OG5_170522	atha|NP_188456	1	-18	45	98
GaLTP31	Type I	OG5_244956	rcom|29742.m001431	2	-9	41	62
GaLTP32	Type I	OG5_244956	rcom|29742.m001431	4	-15	41	80
GaLTP33	Type VI	OG5_156354	rcom|30169.m006486	2	-27	48	97
GaLTP34	Type IV	OG5_150363	rcom|30178.m000847	2	-27	65	98
GaLTP35	Type I	OG5_135466	rcom|29915.m000465	3	-30	56	96
GaLTP36	Type V	OG5_140479	rcom|30143.m001220	8	-42	72	92
GaLTP37	Type I	OG5_156310	rcom|30174.m008761	5	-22	46	99
GaLTP38	Type I	OG5_156310	rcom|30174.m008761	1	-24	48	99
GaLTP39	Type II	OG5_170522	rcom|30066.m000748	1	-9	36	87
GaLTP40	Type II	OG5_170522	rcom|30066.m000748	9	-10	36	87
GaLTP41	Type II	OG5_170522	rcom|30066.m000748	9	-10	36	88
GaLTP42	Type II	OG5_170522	rcom|30066.m000748	4	-10	38	87
GaLTP43	Type II	OG5_170522	rcom|30066.m000748	2	-29	61	99
GaLTP44	Type II	OG5_170522	atha|NP_188456	3	-25	53	100
GaLTP45	Type II	OG5_170522	atha|NP_188456	6	-25	54	100
GaLTP46	Type I	OG5_244956	rcom|29742.m001431	4	-12	35	73
GaLTP47	Type V	OG5_213111	rcom|29780.m001377	1	-40	68	94
GaLTP48	Type I	OG5_135466	rcom|29915.m000465	2	-25	49	97
GaLTP49	Type I	OG5_135466	rcom|29915.m000465	3	-34	58	99
GaLTP50	Type I	OG5_135466	rcom|29915.m000465	5	-34	58	99

GaLTP51	Type V	OG5_213111	rcom|29780.m001377	3	-40	68	94
GhLTP1	Type IX	OG5_212511	rcom|30152.m002431	8	-40	74	94
GhLTP2	Type I	OG5_244785	rcom|27996.m000149	3	-37	75	91
GhLTP3	Type V	OG5_140479	rcom|30143.m001220	3	-41	67	100
GhLTP4	Type I	OG5_212357	atha|NP_187489	2	-22	48	84
GhLTP5	Type I	OG5_135466	rcom|29915.m000465	1	-29	57	96
GhLTP6	Type IV	OG5_150363	rcom|30178.m000847	3	-31	73	82
GhLTP7	Type I	OG5_135466	rcom|29915.m000465	5	-31	56	97
GhLTP8	Type V	OG5_213111	rcom|29780.m001377	3	-40	70	96
GhLTP9	Type VI	OG5_156354	rcom|30169.m006486	5	-28	46	99
GhLTP10	Type V	OG5_140479	rcom|30143.m001220	2	-44	74	96
GhLTP11	Type VI	OG5_244951	rcom|29738.m001007	2	-38	68	98
GhLTP12	Type I	OG5_212196	rcom|29616.m000215	9	-33	64	99
GhLTP13	Type II	OG5_170522	rcom|30066.m000748	4	-10	38	87
GhLTP14	Type II	OG5_170522	rcom|30066.m000748	1	-9	36	87
GhLTP15	Type II	OG5_170522	rcom|30066.m000748	1	-9	36	87
GhLTP16	Type II	OG5_170522	rcom|30066.m000748	3	-10	37	88
GhLTP17	Type II	OG5_170522	rcom|30066.m000748	4	-10	38	87
GhLTP18	Type V	OG5_140479	rcom|30143.m001220	2	-40	66	100
GhLTP19	Type I	OG5_179243	rcom|30005.m001268	6	-27	49	94
GhLTP20	Type II	OG5_170522	rcom|30066.m000748	2	-28	60	99
GhLTP21	Type II	OG5_170522	rcom|30066.m000748	1	-9	36	87
GhLTP22	Type II	OG5_170522	rcom|30066.m000748	4	-10	38	87
GhLTP23	Type II	OG5_170522	rcom|30066.m000748	5	-9	38	87
GhLTP24	Type II	OG5_170522	rcom|30066.m000748	2	-8	32	88
GhLTP25	Type II	OG5_170522	atha|NP_188456	4	-19	47	95

GhLTP26	Type I	OG5_178339	rcom|29983.m003153	9	-23	40	100
GhLTP27	Type IV	OG5_150363	rcom|30178.m000847	2	-30	72	83
GhLTP28	Type I	OG5_135466	osat|NP_001065986	6	-22	44	88
GhLTP29	Type I	OG5_156310	rcom|30174.m008761	8	-25	49	99
GhLTP30	Type VIII	OG5_177605	rcom|30190.m011003	1	-14	41	79
GhLTP31	Type VIII	OG5_177605	rcom|30190.m011003	1	-12	41	80
GhLTP32	Type I	OG5_135466	rcom|29915.m000465	2	-35	59	99
GhLTP33	Type I	OG5_135466	rcom|29915.m000465	8	-32	56	99
GhLTP34	Type I	OG5_135466	rcom|29915.m000465	3	-33	58	99
GhLTP35	Type VIII	OG5_177605	atha|NP_849837	4	-20	52	80
GhLTP36	Type IV	OG5_164277	atha|NP_001032078	9	-17	39	97
GhLTP37	Type IV	OG5_164277	atha|NP_001032078	3	-13	33	99
GhLTP38	Type V	OG5_140479	rcom|30143.m001220	7	-26	48	97
GhLTP39	Type V	OG5_140479	rcom|30143.m001220	1	-25	47	97
GhLTP40	Type V	OG5_140479	rcom|30143.m001220	4	-27	48	97
GhLTP41	Type I	OG5_135466	rcom|29915.m000465	1	-28	52	99
GhLTP42	Type I	OG5_135466	rcom|29915.m000465	1	-29	56	96
GhLTP43	Type I	OG5_244956	rcom|29742.m001431	7	-14	42	74
GhLTP44	Type V	OG5_140479	rcom|30143.m001220	1	-26	48	97
GhLTP45	Type V	OG5_140479	rcom|30143.m001220	2	-29	52	97
GhLTP46	Type II	OG5_170522	atha|NP_188456	5	-19	44	99
GhLTP47	Type I	OG5_135466	osat|NP_001065986	8	-23	46	88
GhLTP48	Type I	OG5_179243	rcom|30005.m001268	5	-27	45	99
GhLTP49	Type II	OG5_170522	atha|NP_188456	2	-24	51	100
GhLTP50	Type II	OG5_170522	atha|NP_188456	1	-23	50	100
GhLTP51	Type II	OG5_170522	atha|NP_188456	2	-23	50	100

GhLTP52	Type II	OG5_170522	atha|NP_188456	1	-22	47	100
GhLTP53	Type II	OG5_170522	atha|NP_188456	1	-25	54	100
GhLTP54	Type I	OG5_178339	rcom|29983.m003153	1	-22	43	87
GhLTP55	Type II	OG5_170522	rcom|30066.m000748	7	-30	61	99
GhLTP56	Type I	OG5_156310	rcom|30174.m008761	6	-21	45	99
GhLTP57	Type V	OG5_140479	rcom|30143.m001220	7	-27	48	97
GhLTP58	Type V	OG5_140479	rcom|30143.m001220	2	-27	50	97
GhLTP59	Type IV	OG5_164277	atha|NP_001032078	9	-17	39	93
GhLTP60	Type IX	OG5_212511	rcom|30152.m002431	2	-39	73	94
GhLTP61	Type III	OG5_178118	rcom|30147.m014294	2	-21	63	77
GhLTP62	Type I	OG5_211688	rcom|30147.m014290	1	-31	59	91
GhLTP63	No group	OG5_170058	rcom|29942.m000735	3	-26	57	75
GhLTP64	Type I	OG5_135466	rcom|29915.m000465	5	-34	58	99
GhLTP65	Type I	OG5_135466	rcom|29915.m000465	4	-34	58	99
GhLTP66	Type II	OG5_170522	rcom|30066.m000748	2	-29	61	99
GhLTP67	Type I	OG5_211688	rcom|30147.m014290	4	-33	58	95
GhLTP68	Type III	OG5_178118	rcom|30147.m014294	3	-21	63	77
GhLTP69	Type V	OG5_213111	rcom|29780.m001377	6	-40	70	98
GhLTP70	Type V	OG5_213111	rcom|29780.m001377	2	-38	66	93
GhLTP71	Type IV	OG5_150363	rcom|29851.m002445	2	-15	52	82
GhLTP72	Type I	OG5_179243	rcom|30005.m001268	2	-27	45	99
GhLTP73	Type II	OG5_170522	atha|NP_188456	3	-25	53	100
GhLTP74	Type II	OG5_170522	atha|NP_188456	2	-24	53	100
GhLTP75	Type I	OG5_135466	rcom|29915.m000465	3	-29	56	96
GhLTP76	Type IV	OG5_150363	rcom|30178.m000847	1	-25	62	95
GhLTP77	Type IV	OG5_150363	rcom|30178.m000847	2	-27	64	98

GhLTP78	Type IV	OG5_150363	rcom|29888.m000322	2	-33	66	100
GhLTP79	Type II	OG5_170522	rcom|30066.m000748	6	-21	52	91
GhLTP80	Type VIII	OG5_177605	rcom|30190.m011003	9	-20	46	89
GhLTP81	Type II	OG5_170522	atha|NP_188456	1	-21	47	100
GhLTP82	Type VIII	OG5_177605	rcom|30190.m011003	3	-13	41	90
GhLTP83	Type VIII	OG5_177605	rcom|30190.m011003	5	-11	38	90
GhLTP84	Type VIII	OG5_177605	rcom|30190.m011003	1	-14	41	85
GhLTP85	Type V	OG5_213111	rcom|29780.m001377	7	-42	70	94
GhLTP86	Type V	OG5_140479	rcom|30143.m001220	3	-44	75	96
GhLTP87	Type IV	OG5_150363	rcom|29888.m000322	2	-31	69	94
GhLTP88	Type I	OG5_244785	rcom|27996.m000149	1	-38	76	85
GhLTP89	Type III	OG5_178118	rcom|30147.m014294	7	-26	71	77
GhLTP90	Type I	OG5_135466	rcom|29915.m000465	3	-25	49	98
GhLTP91	Type VI	OG5_244951	rcom|29738.m001007	4	-33	60	92
GrLTP1	Type IV	OG5_164277	atha|NP_001032078	9	-17	39	93
GrLTP2	Type IV	OG5_150363	rcom|30178.m000847	3	-31	73	82
GrLTP3	Type I	OG5_244785	rcom|27996.m000149	5	-39	77	91
GrLTP4	Type I	OG5_244956	rcom|29742.m001431	3	-15	42	80
GrLTP5	Type V	OG5_140479	rcom|30143.m001220	1	-42	66	100
GrLTP6	Type V	OG5_140479	rcom|30143.m001220	1	-25	49	96
GrLTP7	Type I	OG5_135466	rcom|29915.m000465	1	-28	52	99
GrLTP8	Type V	OG5_213111	rcom|29780.m001377	2	-40	71	98
GrLTP9	Type VIII	OG5_177605	rcom|30190.m011003	2	-14	41	81
GrLTP10	Type VIII	OG5_177605	rcom|30190.m011003	1	-11	40	72
GrLTP11	Type I	OG5_135466	rcom|29915.m000465	5	-31	56	97
GrLTP12	Type V	OG5_140479	rcom|30143.m001220	6	-45	75	96

GrLTP13	Type II	OG5_170522	rcom|30066.m000748	1	-9	38	89
GrLTP14	Type II	OG5_170522	rcom|30066.m000748	1	-10	39	87
GrLTP15	Type IV	OG5_150363	rcom|30178.m000847	7	-27	63	98
GrLTP16	Type II	OG5_170522	atha|NP_188456	2	-15	55	68
GrLTP17	Type V	OG5_140479	rcom|30143.m001220	2	-41	67	100
GrLTP18	Type III	OG5_178118	rcom|30147.m014294	2	-24	68	76
GrLTP19	Type I	OG5_178339	rcom|29983.m003153	7	-23	41	100
GrLTP20	Type I	OG5_156310	rcom|30174.m008761	7	-25	49	99
GrLTP21	Type V	OG5_140479	rcom|30143.m001220	1	-26	48	97
GrLTP22	Type V	OG5_140479	rcom|30143.m001220	2	-26	48	97
GrLTP23	Type VI	OG5_244951	rcom|29738.m001007	2	-38	68	92
GrLTP24	Type I	OG5_212196	rcom|29616.m000215	4	-33	69	95
GrLTP25	Type IV	OG5_164277	atha|NP_001032078	2	-17	36	99
GrLTP26	Type I	OG5_135466	osat|NP_001065986	1	-21	45	86
GrLTP27	Type I	OG5_135466	osat|NP_001065986	1	-21	45	86
GrLTP28	Type IV	OG5_150363	rcom|29888.m000322	2	-32	70	94
GrLTP29	Type VI	OG5_156354	rcom|30169.m006486	1	-24	47	92
GrLTP30	Type I	OG5_244956	rcom|29742.m001431	3	-12	35	73
GrLTP31	Type IX	OG5_212511	rcom|30152.m002431	1	-39	74	94
GrLTP32	Type IV	OG5_150363	rcom|29851.m002445	4	-16	52	82
GrLTP33	Type V	OG5_213111	rcom|29780.m001377	6	-39	67	93
GrLTP34	Type II	OG5_170522	rcom|30066.m000748	3	-28	64	91
GrLTP35	Type I	OG5_211688	rcom|30147.m014290	6	-33	58	95
GrLTP36	Type II	OG5_170522	rcom|30066.m000748	2	-21	53	91
GrLTP37	Type I	OG5_179243	rcom|30005.m001268	4	-27	47	98
GrLTP38	Type I	OG5_212357	atha|NP_187489	1	-23	52	83

GrLTP39	Type I	OG5_135466	rcom|29915.m000465	3	-29	56	96
GrLTP40	Type I	OG5_135466	rcom|29915.m000465	3	-33	58	99
GrLTP41	Type I	OG5_135466	rcom|29915.m000465	8	-32	56	99
GrLTP42	Type I	OG5_135466	rcom|29915.m000465	7	-34	58	96
GrLTP43	Type I	OG5_135466	rcom|29915.m000465	1	-25	50	97
GrLTP44	Type II	OG5_170522	rcom|30066.m000748	5	-30	61	99
GrLTP45	Type II	OG5_170522	atha|NP_188456	6	-24	51	100
GrLTP46	Type II	OG5_170522	atha|NP_188456	2	-24	51	100
GrLTP47	Type II	OG5_170522	atha|NP_188456	9	-26	54	100

Table S4: Ka/Ks analysis for orthologous gene pairs of *G. hirsutum, G. arboreum and G. raimondii*.

	Orthologous gene pairs		Subfamily	Ka	Ks	Ka/Ks	Age (MYA)
	GhLTP18	*GaLTP4*	Type V	1.04349	0.886609	1.17695	170.5017308
	GhLTP27	*GaLTP5*	Type IV	1.17407E-08	0.0117407	0.000001	2.257826923
	GhLTP38	*GaLTP20*	Type V	0.0069053	0.000138106	50	0.026558846
	GhLTP39	*GaLTP19*	Type V	0.00825326	0.0087886	0.939087	1.690115385
	GhLTP40	*GaLTP18*	Type V	2.47973E-07	6.3304E-07	0.391718	0.000121738
	GhLTP42	*GaLTP35*	Type I	0.00430393	0.00992967	0.433442	1.909551923
	GhLTP53	*GaLTP12*	Type II	0.0613213	0.0385255	1.59171	7.40875
	GhLTP54	*GaLTP1*	Type I	0.00881441	0.02759	0.319478	5.305769231
	GhLTP56	*GaLTP37*	Type I	0.00427851	0.00950197	0.450276	1.827301923
	GhLTP64	*GaLTP49*	Type I	0.031233	0.0216431	1.4431	4.162134615
	GhLTP65	*GaLTP50*	Type I	0.00351433	0.0138141	0.254401	2.656557692
A_t vs A	*GhLTP66*	*GaLTP43*	Type II	2.25862E-07	6.50533E-07	0.347195	0.000125103
	GhLTP71	*GaLTP23*	Type IV	0.0126478	0.0133806	0.945236	2.573192308
	GhLTP73	*GaLTP44*	Type II	2.53972E-07	5.76818E-07	0.440298	0.000110927
	GhLTP74	*GaLTP12*	Type II	0.00455055	9.10111E-05	50	0.017502135
	GhLTP82	*GaLTP22*	Type VIII	1.08223	0.737177	1.46807	141.7648077
	GhLTP83	*GaLTP22*	Type VIII	1.09202	0.706215	1.5463	135.8105769
	GhLTP87	*GaLTP28*	Type IV	0.009919	0.0117401	0.844878	2.257711538
	GhLTP10	*GaLTP36*	Type V	0.0336001	0.0220565	1.52336	4.241634615
	GhLTP46	*GaLTP26*	Type II	0.00889998	0.000178	50	0.034230769
	GhLTP69	*GaLTP25*	Type V	0.0119217	0.000238434	50	0.045852692
	GhLTP86	*GaLTP36*	Type V	0.0251262	0.0108128	2.32375	2.079384615
	GhLTP90	*GaLTP48*	Type I	0.00375983	0.0113541	0.331142	2.183480769

	GhLTP1	*GrLTP31*	Type IX	0.00426027	8.52054E-05	50	0.016385654
	GhLTP2	*GrLTP3*	Type I	0.00855098	0.00017102	50	0.032888462
	GhLTP3	*GrLTP17*	Type V	0.00425852	0.00961733	0.442797	1.849486538
	GhLTP6	*GrLTP2*	Type IV	0.00429226	8.58451E-05	50	0.016508673
	GhLTP9	*GrLTP29*	Type VI	1.06915	0.79499	1.34486	152.8826923
	GhLTP12	*GrLTP24*	Type I	0.982879	1.04509	0.940469	200.9788462
	GhLTP22	*GrLTP14*	Type II	0.00507016	0.0108497	0.467308	2.086480769
	GhLTP29	*GrLTP20*	Type I	0.00417259	0.0201059	0.20753	3.866519231
	GhLTP30	*GrLTP9*	Type VIII	1.05288	0.823327	1.27882	158.3321154
	GhLTP32	*GrLTP42*	Type I	1.01866	0.943358	1.07983	181.415
	GhLTP33	*GrLTP41*	Type I	9.00048E-09	0.00900048	0.000001	1.730861538
D_t vs D	*GhLTP34*	*GrLTP40*	Type I	0.00357905	0.000071581	50	0.013765577
	GhLTP37	*GrLTP25*	Type IV	1.00203	0.993626	1.00846	191.0819231
	GhLTP41	*GrLTP7*	Type I	0.0117437	0.0210766	0.557194	4.053192308
	GhLTP48	*GrLTP37*	Type I	1.00258	0.99304	1.00961	190.9692308
	GhLTP55	*GrLTP44*	Type II	0.00455461	0.0180979	0.251665	3.480365385
	GhLTP70	*GrLTP33*	Type V	0.0107628	0.0182325	0.590311	3.50625
	GhLTP77	*GrLTP15*	Type IV	0.0466742	0.0441638	1.05684	8.493038462
	GhLTP79	*GrLTP36*	Type II	0.00478796	0.0268971	0.17801	5.172519231
	GhLTP11	*GrLTP23*	Type VI	0.0345295	0.0122443	2.82004	2.354673077
	GhLTP67	*GrLTP35*	Type I	0.00384047	0.0112046	0.342759	2.154730769
	GhLTP76	*GrLTP15*	Type IV	0.0269784	0.10365	0.260284	19.93269231
	GhLTP78	*GrLTP28*	Type IV	1.93462E-07	6.99423E-07	0.276602	0.000134504

Table S5: PCR primers used for qRT-PCR in this study.

Gene name	Forward primer (5'-3')	Reverse primer (5'-3')
GaLTP3	CTCCTACTGGCCGAAGCAAA	GGCAAGGCTTTTGCTCTTTGA
GaLTP5	TGGGTTTAACGGTGGAAGGG	GCTCCTTTAAGAGCCTCGCA
GaLTP9	CTTGCAAGCCGGTGGTTTAC	GCAAACGCATTCCAGATGGG
GaLTP22	TTGGGGACGAAGATAGCGAC	GCAGCCACGAGTGATCCTAA
GaLTP25	TGGGAACGCAAAGGCAAAAG	ACAAAGGCACCTCGGAGAAG
GaLTP28	CATGCAAACCAGCAGTGACC	GGAAGGCAAGAGCTTGGAGT
GaLTP29	CTTGTGTGCGGTGGCACTA	CATGGGATGAGCTCGATGGG
GaLTP34	GCTCTGAAAGGGGCTGACTT	TGGAGGCAAATCCAAGGCAA
GaLTP35	AAGTGGCCGGTTGCTTATCA	CGAGCCATGTTGTTCAGGGA
GaLTP36	GTCATGCTTTCCAACACGGC	CACTGGTAACCAACAGGACGA
GaLTP44	CAGGCTGAAGGAACAACAGC	TGGGACTCCACAAGTCGAAG
GaLTP47	AAGGTGGGTGCCTTACTCAG	ATGATCCCGGCCTGCTTTG
GaLTP48	AGGGGCCATAAGCTGTGTTC	GGTCTAGTCTTCTTCGGGC
GaLTP49	GGACTGGTGCTGGTGCTATT	CACTTGACGCTGTTGCAGTC
GaLTP50	AGATGCATCAAAAGTGCGGC	CACTTGACGCTGTTGCAGTC
GaLTP51	TGGGTGCCTTACTCAGGACT	CATGATCCCGGCTTGCTTTG
GrLTP1	GGAGCTAAAGGGGCAACCAT	CCCAAGAACTTACCCCTGCAT
GrLTP2	CTGTCGACCCAACACCTCAA	CTGGTAAACCCATGGCGAGT
GrLTP8	TGGGAACGCAAAGGCAAAAG	ACAAAGGCACCTCGGAGAAG
GrLTP10	TTCAACAACGCCACTCTCCA	TTATCAGTGACACCGCAGCC
GrLTP11	AAGTGGCCGGTTGCTTATCA	ACACTACATTTGGCCGGGAG
GrLTP12	GTCATGCTTTCCAACACGGC	CACTGGTAACCAACAGGACGA
GrLTP13	CGTGGTGACTCTAGTGGTGG	GCTGATGGAGGTGGCTTCTT
GrLTP14	TTTCGGGTGCAGAGACAACG	CAGCTGATGGAGGTGGCTTC
GrLTP21	TTCTGAGCGTTGCTGTGCTA	ACCAACAGGACGAACTGCAA
GrLTP22	TCCCCATGTACATACGCAGC	GCAGAGGCAGTCTGGATTGT
GrLTP23	CTTGCAAGCCGGTGGTTTAC	GCAAACGCATTCCAGATGGG
GrLTP25	GTTGCTTATTGCAGCGGTGG	GCTTGCACTGCTCCAACTTT
GrLTP28	TTGCAACATGCCAGCTTCAG	GGAAGGCAAGAGCTTGGAGT
GrLTP33	AAGGTGGGTGCCTTACTCAG	ATGATCCCGGCCTGCTTTG
GrLTP34	ATACGGCGGTATTCTCAGCG	ATGAGCTCAGCTGTGTTGGG
GrLTP40	CTGCGGCGGCATAAAATCTC	TGTTGCAGTCAGTGCTAGGG
GrLTP41	CTGTGGTCAAGTCACAGGCT	TGGGAGTCCGCTTGCAATAC
GrLTP43	CTCAAGGGGCCATAAGCTGT	TATTCGGGCGGCAGATTTGA
GrLTP47	TTTTCGGGTGAAAGTCGCAC	GAGGCGAGGACGACGTAAG
GhLTP1	TGCTGCTTCTTTTTGGGTCC	TCACTTGGTGGAATACGGCT
GhLTP6	CTGTCGACCCAACACCTCAA	CTGGTAAACCCATGGCGAGT
GhLTP7	AAGTGGCCGGTTGCTTATCA	ACACTACATTTGGCCGGGAG
GhLTP8	CTTCTCCGAGGTGCCTTTGT	TGTCTCGATCTTGTTTACTTCCACA
GhLTP9	TGAGGTTGGCAGTGTTGATGA	TTGCCCGCATTGGGATATGA
GhLTP10	GTCATGCTTTCCAACACGGC	CACTGGTAACCAACAGGACGA
GhLTP11	CTTGCAAGCCGGTGGTTTAC	GCAAACGCATTCCAGATGGG

GhLTP12	TTCTGCTGTTGATTGCCCCA	TAGTTCACGCAAGGCCTCAG
GhLTP18	GCCTGGGAAAGGTGGTTGAT	ACAGGGGCATTCTCGTCTTG
GhLTP20	CTCCTACTGGCCGAAGCAAA	GGCAAGGCTTTGCTCTTTGA
GhLTP27	TGGGTTTAACGGTGGAAGGG	GCTCCTTTAAGAGCCTCGCA
GhLTP28	CCTGGACTCAACTACGACCG	TGGAGCAATCGACATCAGGG
GhLTP30	AACAAGCCGTCGCTACTGAA	TGATGGCCAGGAACACTGAC
GhLTP31	TTCAACAACGCCACTCTCCA	TTATCAGTGACACCGCAGCC
GhLTP33	CTGTGGTCAAGTCACAGGCT	TGGGAGTCCGCTTGCAATAC
GhLTP34	CTGCGGCGGCATAAAATCTC	TGTTGCAGTCAGTGCTAGGG
GhLTP38	GGCTAAACGGAGTGGAAGCA	TAGCACAGCAACGCTCAGAA
GhLTP40	CCGGTTTCTGAGCGTTGTTG	AAATGGGAAACCTCCGCACT
GhLTP41	TCTTGTGCTTGGTTGTGGGT	CAGCACCGTTGTTTCTCACG
GhLTP42	AAGTGGCCGGTTGCTTATCA	CGAGCCATGTTGTTCAGGGA
GhLTP44	TCAACGGAGTGGAAGCAACT	ATAGCACAGCAACGCTCAGA
GhLTP47	TCCATGTTGTTGCCTCGTCA	ACCCGTACAGCAACTTGGAG
GhLTP50	GCTGAAGGAACACCAGCCAT	CGAAGCAACCCTTTTAGCGT
GhLTP53	TTTTCGGGTGAAAGTCGCAC	GAGGCGAGGACGACGTAAG
GhLTP58	AAAGCTCAGCAATCCAGGCT	TTGGAATGGTGACGGCAACT
GhLTP59	GGAGCTAAAGGGGCAACCAT	CCCAAGAACTTACCCCTGCAT
GhLTP60	CCATCAAAGCTCTGGGGCAA	GGCCATGTTCCGATCCACA
GhLTP61	GTGCAGACCCGAACGAGTAA	GGGAATTAGGCTGGGTCTCG
GhLTP65	AGATGCATCAAAAGTGCGGC	CACTTGACGCTGTTGCAGTC
GhLTP69	CCTTGTTGCACTGGTTGTGT	CCGCAGCAGACCTAAATGGA
GhLTP70	AAGGTGGGTGCCTTACTCAG	ATGATCCCGGCCTGCTTTG
GhLTP73	CAGGCTGAAGGAACAACAGC	TGGGACTCCACAAGTCGAAG
GhLTP74	GAAGCCATGCCTCTGTGGAT	CTCCACAAGTCGAAGCAACG
GhLTP79	GTGTGTGGTGGGGATAGTGG	GGAGGCTTCGACGACGTAAT
GhLTP81	CGGAGGCAGTGACTTGTGAT	AAGCATGGCTTCTGCTCTGT
GhLTP82	GGATCCGACTTGTTTGCAGC	TCTGATCAGTGACACCGCAG
GhLTP83	CAGCTTGAAGTCTTGCGCTC	GCAGCCACGAGTGATCCTAA
GhLTP85	TGGGTGCCTTACTCAGGACT	CATGATCCCGGCTTGCTTTG
GhLTP86	CCAGAATCCGCGATGCCTAT	GCAATGTTGCAGCGTTTTGG
GhLTP87	CATGCAAACCAGCAGTGACC	TACCAAGGGAAGGCAACAGC
GhLTP89	TGAGCTCACCAACTTGAATGTG	CCACAAGTCAGAGGAGGCAG
GhLTP90	AGGGGCCATAAGCTGTGTTC	GGTCTAGTCTTCTTCGGGC
GhLTP91	GCATGCAAGGGCGTGATAAG	ATGGCGAGGGACTTGTCTTC
